# Barriers to the Intestinal Absorption of Four Insulin-Loaded Arginine-Rich Nanoparticles in Human and Rat

**DOI:** 10.1021/acsnano.2c04330

**Published:** 2022-08-23

**Authors:** Patrik Lundquist, Georgiy Khodus, Zhigao Niu, Lungile Nomcebo Thwala, Fiona McCartney, Ivailo Simoff, Ellen Andersson, Ana Beloqui, Aloise Mabondzo, Sandra Robla, Dominic-Luc Webb, Per M. Hellström, Åsa V Keita, Eduardo Sima, Noemi Csaba, Magnus Sundbom, Veronique Preat, David J. Brayden, Maria Jose Alonso, Per Artursson

**Affiliations:** † Department of Pharmacy, Uppsala University, SE-751 43 Uppsala, Sweden; ‡ Department of Pharmacy and Pharmaceutical Technology, CIMUS, Universidade de Santiago de Compostela, Santiago de Compostela ES 15782, Spain; § Université catholique de Louvain, UCLouvain, Louvain Drug Research Institute, Advanced Drug Delivery and Biomaterials, BE 1200 Brussels, Belgium; ∥ UCD School of Veterinary Medicine, University College Dublin, Belfield D04 V1W8, Ireland; ⊥ Department of Surgery in Norrköping, Linköping University, SE-581 83 Norrköping, Sweden; # Department of Biomedical and Clinical Sciences, Linköping University, SE-581 83 Linköping, Sweden; ∇ CEA, Institute of Biology and Technology of Saclay, Department of Pharmacology and Immunoanalysis, Gif sur Yvette FR 91191, France; ○ Department of Medical Sciences, Uppsala University, SE-751 85 Uppsala, Sweden; ◆ Department of Surgical Sciences−Upper Abdominal Surgery, Uppsala University, SE-751 85 Uppsala, Sweden

**Keywords:** nanoparticle, insulin, oral peptide delivery, jejunum, human

## Abstract

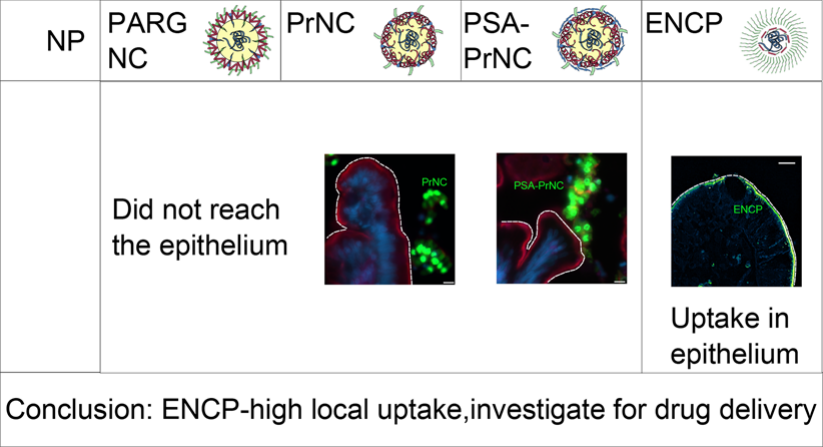

Peptide drugs and biologics provide opportunities for treatments of many diseases. However, due to their poor stability and permeability in the gastrointestinal tract, the oral bioavailability of peptide drugs is negligible. Nanoparticle formulations have been proposed to circumvent these hurdles, but systemic exposure of orally administered peptide drugs has remained elusive. In this study, we investigated the absorption mechanisms of four insulin-loaded arginine-rich nanoparticles displaying differing composition and surface characteristics, developed within the pan-European consortium TRANS-INT. The transport mechanisms and major barriers to nanoparticle permeability were investigated in freshly isolated human jejunal tissue. Cytokine release profiles and standard toxicity markers indicated that the nanoparticles were nontoxic. Three out of four nanoparticles displayed pronounced binding to the mucus layer and did not reach the epithelium. One nanoparticle composed of a mucus inert shell and cell-penetrating octarginine (ENCP), showed significant uptake by the intestinal epithelium corresponding to 28 ± 9% of the administered nanoparticle dose, as determined by super-resolution microscopy. Only a small fraction of nanoparticles taken up by epithelia went on to be transcytosed via a dynamin-dependent process. *In situ* studies in intact rat jejunal loops confirmed the results from human tissue regarding mucus binding, epithelial uptake, and negligible insulin bioavailability. In conclusion, while none of the four arginine-rich nanoparticles supported systemic insulin delivery, ENCP displayed a consistently high uptake along the intestinal villi. It is proposed that ENCP should be further investigated for local delivery of therapeutics to the intestinal mucosa.

Peptide and protein drugs promise treatments for many diseases but also pose challenges that are not encountered by traditional small molecule drugs.^1−3^ Many of these challenges are related to the molecules’ size and polarity, which limit their capacity to pass cell and tissue barriers and reach the intended target. Another major problem is their vulnerability to chemical and enzymatic degradation. Delivery of peptides to the systemic circulation via the oral route has so far been largely unsuccessful, due to degradation in the gastrointestinal (GI) tract and poor permeation across the intestinal wall.^1−3^ These drawbacks often result in low and highly variable bioavailability of peptides. To circumvent such obstacles, parenteral routes are the major routes of administration for biologics, as well as peptide drugs. At present five oral peptide drugs are marketed, cyclosporin A, voclosporin, desmopressin, octreotide, and semaglutide, where only semaglutide displays a size similar to insulin.^4^ Evidence for successful local delivery of peptide drugs to the intestinal mucosa (e.g., for treatment of inflammatory bowel disease) is also limited but is a lower hurdle. Nevertheless, oral administration of peptides remains a major research field since successful oral delivery would result in higher patient compliance and reduced inconvenience and costs and, for liver targeted peptides, can give a more physiologically relevant response with fewer side effects than current injection strategies.^3,4^ Delivery strategies for improved oral absorption of peptides include peptide stabilization, coadministration with peptidase inhibitors and/or permeation enhancers, use of patch and microneedle devices, and incorporation into nanoparticle delivery systems (NP).^1,2^

NP for oral delivery of peptides have been investigated since the 1980s.^5^ The idea is that NP will protect peptide drugs from degradation in the gastrointestinal tract and enhance their delivery across the intestinal wall through epithelial uptake of the peptide-loaded NP.^6−8^ So far, these attempts have largely been unsuccessful. Promising results in small animals have been obtained in some cases, but follow up studies in larger species including humans are scarce or disappointing.^1,2^

A reason for the disappointing results is most likely related to the fact that many elegantly designed nanoparticle formulations are not well characterized with regard to key pharmaceutical requirements, such as ability to protect or to carry a sufficient payload of their sensitive peptide cargo, the interaction of NPs with intestinal tissues, as well as difficulties in making reproducible formulations that can be scaled up for manufacture. Furthermore, knowledge about the toxicity of these formulations is often rudimentary.

In an attempt to address these and related issues, the pan-European collaborative project TRANS-INT was formed in 2012 with the aim to investigate a wide variety of NP compositions as vehicles for the oral delivery of peptide drugs.^2,9^ Several hundred NP covering a broad range of chemical and physical properties developed by leading nanotechnology groups in Europe were studied.

NP were systematically evaluated, all according to the same protocols, according to predefined specifications developed in collaboration with partners from the pharmaceutical industry. The most successful candidates were selected for study of NP–tissue interactions in *ex vivo* and *in vivo* models. The goal was to define the suitability of each NP as an oral delivery system for peptides and proteins using insulin as a model, including NP interactions with the intestinal mucosa *ex vivo* and *in vivo* (Figure 1A). A goal of TRANS-INT was also to ensure a complete reporting of methods and results allowing reproduction of data; such criteria have now been formalized as the MIRIBEL criteria (Minimum Information Reporting in Bio–Nano Experimental Literature), to which the present study conforms.^10^

**Figure 1:**
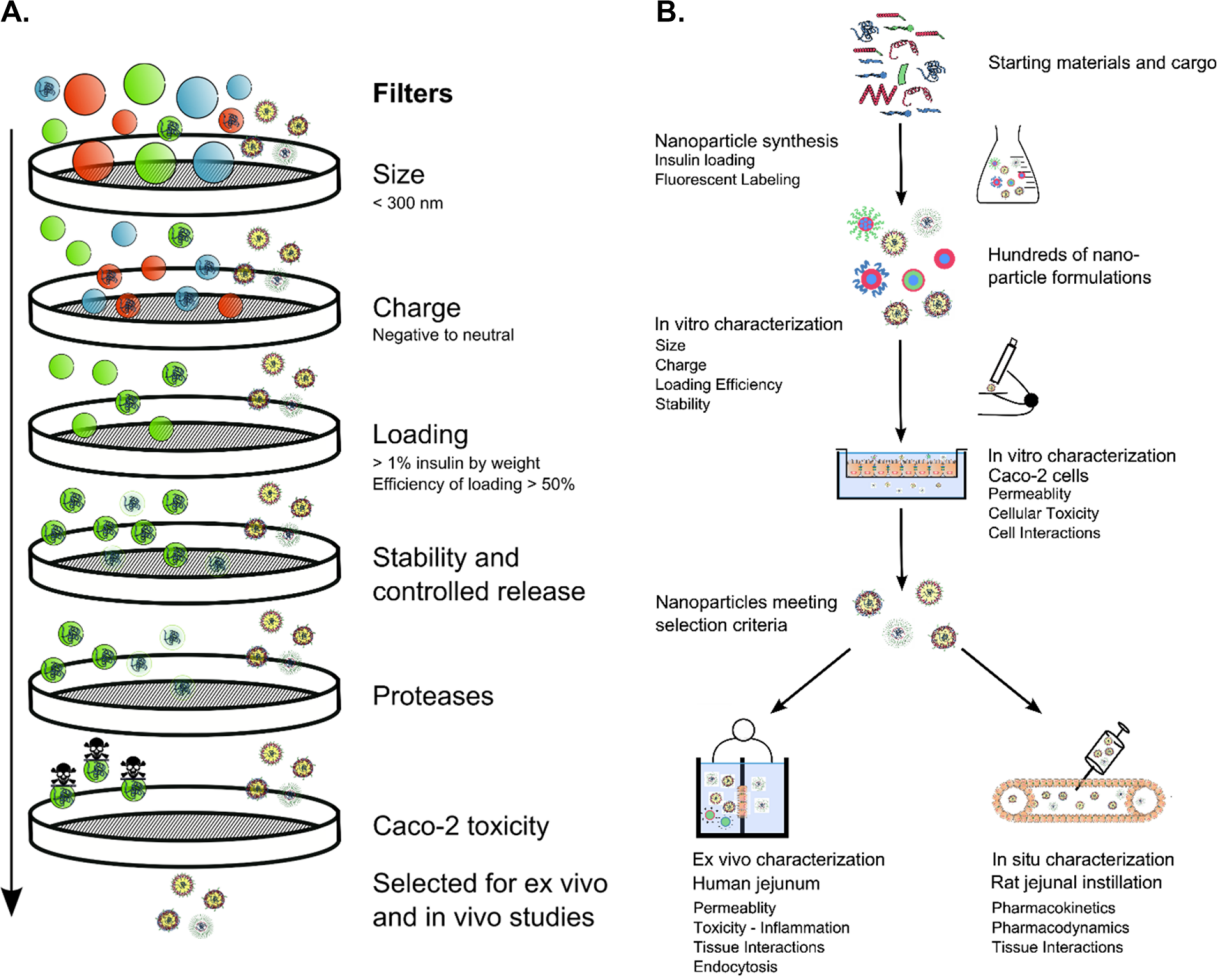
A. TRANS-INT NP evaluation criteria. NP were prioritized according to criteria of size (<350 nm diameter), negative or approximately neutral surface charge (ζ-potential), loading efficiency (incorporation of more than 50% of added peptide), final loading of peptide (>1% by weight), stability of formulation with regard to dissolution and stability in simulated intestinal fluids, relevant buffers, cell culture media, and controlled release of peptide cargos in these media.^2,9^ NP passing these criteria were evaluated in initial *in vitro* tests including protection of cargo against proteases, and toxicity in Caco-2 cell culture. After evaluation, four TRANS-INT NP were selected for further *in vitro*, *ex vivo* and *in situ* evaluation in this study.^11−13^ (B) Study workflow. Arginine-rich NP selected for inclusion in this study were resynthesized and evaluated according to TRANS-INT criteria. NP components were fluorescently labeled. NP permeability and interactions with Caco-2 cells were investigated. NP were characterized in isolated human jejunal tissues mounted in an Ussing chamber *ex vivo*, and in rat jejunal loops *in situ*. Permeability and tissue interactions of the NP were investigated, and PKPD of the insulin formulation was monitored in the rat circulation.

NP were prioritized according to a number of criteria as detailed in Figure 1A. NP passing the initial hurdles were subjected to *in vitro* tests, including protection of cargo against proteases (for more than 3 h), mucus binding and muco-permeation assays, and cytotoxicity in Caco-2 cell culture.^2,9^ Testing of multiple NP batches across several independent reference laboratories ensured reliability and reproducibility of results. Together, these tests provided stringent prioritization criteria for the selection of NPs for more advanced evaluation in small animals *in vivo* and in human intestinal tissue *ex vivo*. The attrition rate was high. Out of 13 NP prototype families, in total comprising several hundred NP formulations, 5 NP progressed to *ex vivo* studies with human tissue.^9^ The four TRANS-INT NP prototypes selected for our study came from three related NP prototype families and emerged after prioritization according to these quality criteria.^11−13^ All four NP contained arginine-rich oligopeptides or polypeptides with potential to interact with cell membranes and possibly to enable NP penetration through the plasma membrane.^11−15^ This presents a delicate balance: the positive charge of the arginine-containing peptides is essential for membrane interaction and penetration, but an NP with a high positive charge will likely be immobilized in the mucus layer.^1,16−18^ When the arginine-rich peptides form complexes with the negatively charged insulin, the net charge will be reduced. By aiming for an almost electrically neutral NP, it was hoped that a balance would be struck, exposing enough arginine residues to enable membrane penetration while avoiding mucus binding.^19,20^ Furthermore, different surface coatings of the NP were tested in an attempt to minimize interactions with the intestinal mucus layer without inhibition of the essential interaction with the intestinal epithelium. NP were loaded with either insulin glulisine or Insuman (human insulin), provided by Sanofi (Frankfurt, Germany). The following four NP were selected for this study: (a) polyarginine (PARG)–oleic acid–insulin nanocapsules (PARG NC);^11^ (2) protamine nanocomplexes (PrNC);^12,14^ (3) polysialic-acid-covered PrNC (PSA-PrNC);^12,14^ and (4) polyglutamic acid–polyethylene glycol (PGA–PEG)-enveloped lauric acid–octarginine (C12-R8) insulin nanocomplexes (ENCP).^13^ The detailed design and formulation of these NP have been presented elsewhere.^11−13^

The NP batches were produced exclusively for this study and were characterized as detailed in Figure 1 prior to the investigations in the human jejunal tissues *ex vivo* and rat intestine *in situ*. NP were then investigated in freshly isolated human jejunal tissue mounted in Ussing chambers.^1^ This set up contains the barriers encountered by peptide drugs and NP after administration *in vivo*, including an intact native mucus layer, proteolytic enzymes in the mucus and epithelium, the epithelial cell monolayer, and the underlying lamina propria.^1,21^ Permeability and tissue toxicity of the NP were quantified. NP localization in the tissue was identified, and the number of nanoparticles taken up by the tissue was quantified using super-resolution microscopy (structured illumination microscopy, SIM).^22^ The investigations in human jejunum were complemented with studies *in situ* in rat intestinal loops, where NP–tissue interactions as well as pharmacokinetics and pharmacodynamics (PKPD) of the insulin cargo and its effect on blood glucose were investigated.^21,23,24^ The workflow of our study is illustrated in Figure 1B.

## Results and Discussion

### NP Composition and Characteristics

Electron micrographs of the NP included in the study are shown in Figure 3. Additionally, schematics of a basic core–shell structure for the four arginine-rich NP (PARG NC, PrNC, PSA-PrNC, and ENCP) are illustrated. Cationic and anionic charged polystyrene nanoparticles (PS-NP+ and PS-NP−) were included as reference nanoparticles (without cargo). The four TRANS-INT NP were loaded with insulin glulisine or Insuman. Loading, size, ζ-potential, and other characteristics of the NP were determined and are listed in Table 1. The NP characteristics were in agreement with previously published data on these NP.^11−14^ NP sizes ranged from 178 to 339 nm while ζ-potentials ranged from −34 to +2 mV. PS-NP+, and PS-NP– controls extended this range from −48 to +24 mV. In accordance with selection criteria (Figure 1A) the four NP all showed adequate stability during storage as well as in simulated intestinal fluids, buffers, and cell culture media, and they protected their cargo from enzymatic degradation, in line with previously published data.^11−14^

**Table 1:**
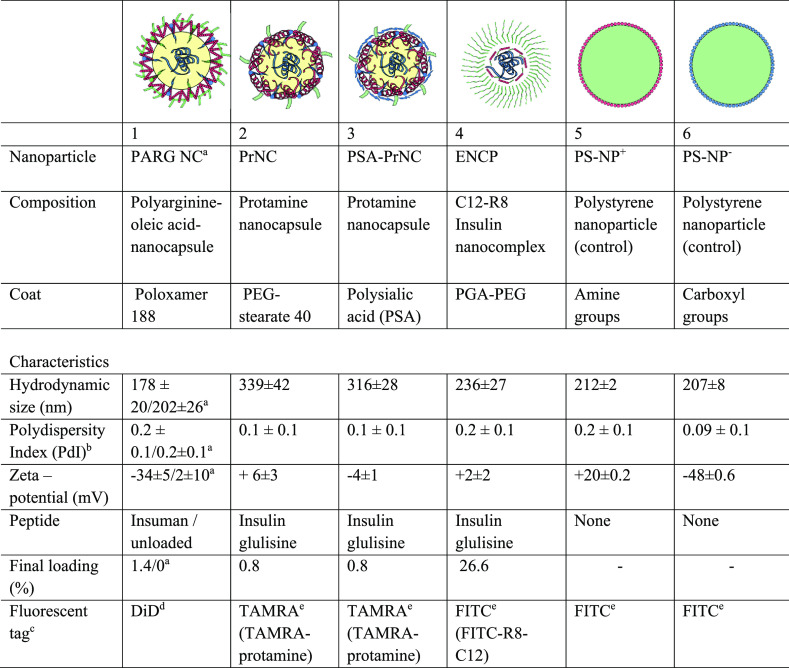
Characterization of NP^f^^,^^g^

a Values for insulin-loaded PARG NC/unloaded PARG NC; PARG NC was used in unloaded form in Ussing chamber experiments.

b PdI values reported are average values from DLS measurements.

c Tetramethylindodicarbocyanine (DiD); fluorescein isothiocyanate (FITC); tetramethylrhodamine (TAMRA).

d Fluorescent tag dissolved in NP lipid phase.

e Fluorescent tag covalently attached.

f Further data on nanoparticle stability and *in vitro* characterization can be found in refs (10−14).

g Values are given as average ± SD.

**Figure 2:**
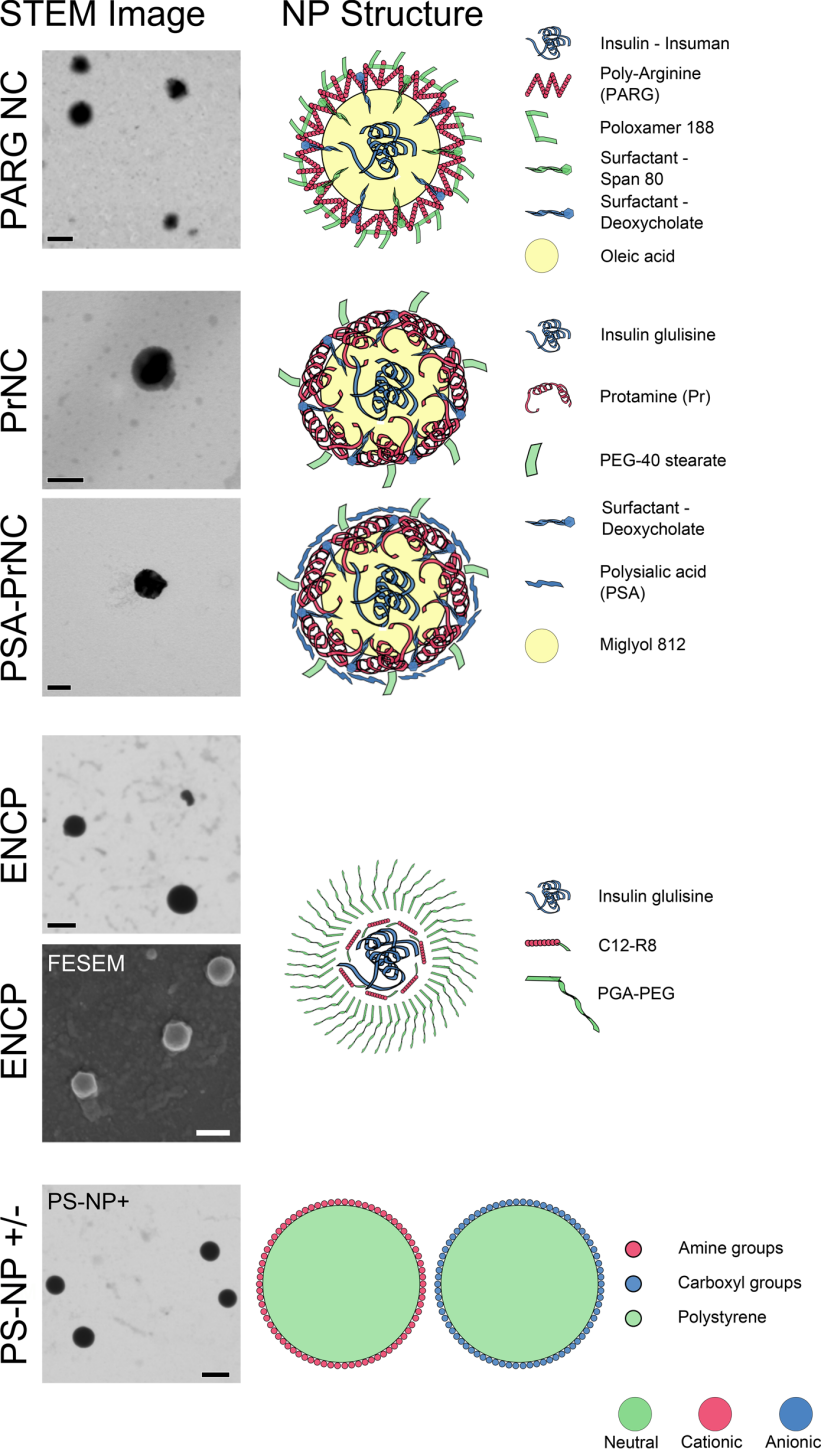
Schematics and micrographs of proposed NP structures. Electron micrographs of the included NP are shown. Sketches of NP structures are illustrations of NP composition and not exact models. The sketches depict a basic model of the NP core–shell structure indicating the localization of insulin and the assumed composition of the NP surface. Actual structures are likely to be more complex and dynamic. Components are not to scale. NP components are color-coded by charge. Fluid oil phases are indicated in yellow. Scale bars in black or white represent 200 nm. Abbreviations: STEM, scanning transmission electron microscopy; FESEM, field emission scanning electron microscopy.

The surface coating of the NP influences interactions with the intestinal mucus layer. Steric stabilization by surface PEGylation has been investigated as a strategy to reduce mucus binding of NP.^6,19,25^ It was reasoned that PEG groups incorporated in the poloxamer 188 coating of the PARG NC might improve mucus penetration of the NP. The surface of PrNC was also coated with PEG-stearate-40, for the same purpose. Similarly, it was reasoned that addition of an outer layer of polysialic acid (PSA) to PrNC to form PSA-PrNC could further improve mucus penetration, since PSA is a natural hydrophilic constituent of mucus.^26^ Additionally, the PSA coating protected the insulin cargo against proteolytic breakdown.^12^ ENCPs were designed with a dense coat of short PEG chains, creating an electrically neutral, highly hydrophilic, and muco-diffusive surface on the NP. The PEG coat of the ENCP was theoretically denser than those of the other arginine-rich NP.^3,11^ In reality, the NP core–shell structure proposed here might be a simplification, and the actual NP structure might be both more complex and dynamic. The sketches of the NP in Figure 2 are illustrations, and the individual components are not to scale.

Poly- and oligo-arginines can, due to their positive charge, form insulin complexes and interact with the negatively charged phospholipids in the plasma membrane. The poly-l-arginine in the PARG NC has a *M*_w_ of 27–36 kDa and can aid interactions with the membrane but has not yet been demonstrated to enhance membrane permeation. However, some studies indicate that poly-l-arginine can act as a permeation enhancer and transiently increase epithelial permeability by opening paracellular tight junctions.^15^ Salmon protamine (*M*_w_ 372 Da) included in PrNC and PSA-PrNC as well as in approved insulin formulations has been reported to act as a cell-penetrating peptide (CPP),^27^ and as a permeation enhancer.^28^ This can be explained by arginine-rich sequences in the protamine peptide sharing homology to the canonical cell-penetrating peptide, Trans-Activator of Transcription (TAT).^17^ ENCP (Figure 2) included C12-R8, octarginine (R8) linked to lauric acid (C12), a medium chain fatty acid.^15^ R8 is a cell-penetrating peptide derived from the TAT sequence that has been reported to aid insulin intestinal permeability when complexed with it.^29^ C12 is also an intestinal epithelial permeation enhancer, and it was included to aid R8-mediated epithelial permeation.^30,31^ Additionally, C12-R8 was found to show superior complexation of insulin when compared with R8, presumably due to a combination of ionic and hydrophobic interactions, leading to a reproducible formation of stable nanocomplexes.^13^

### NP Permeability Across Human Jejunum *ex Vivo*

For permeability and visualization experiments, NP were labeled with fluorescent tags. Labeled NP showed a wide range of permeabilities across human intestinal tissue (Table 2). PARG NC, as well as the two reference polystyrene NP controls (PS-NP+ and PS-NP−), had similar and very low to undetectable permeabilities. In contrast to these, three NP, PrNC, PSA-PrNC, and ENCP, showed 100-fold higher permeabilities. *P*_app_ values for these three NP were however still below 10^–7^ cm/s (Table 2). When permeability values for these three NP were scaled to a predicted jejunal fraction absorbed, using a basic PBPK model of jejunal absorption in man,^32^ these *P*_app_ values (Table 2) corresponded to a predicted jejunal absorption of less than 0.5% of the administered dose (Table 2).

**Table 2:**
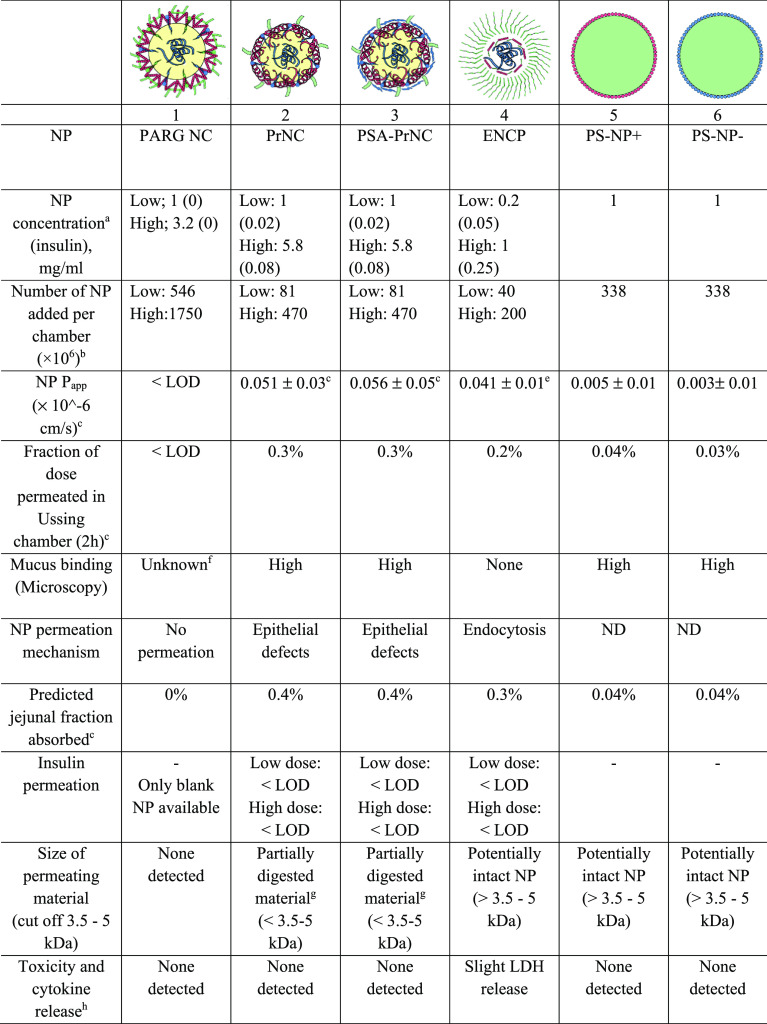
NP Permeability and Tissue Interaction Data from Ussing Chamber Experiments with Human Jejunum^i^

a Initial Ussing permeability experiments with NPs were performed at NP concentrations found to be nontoxic in Caco-2 cells. When no toxicity was found at this concentration, NP concentrations were then increased to the high value. The resulting concentration of insulin in mg/mL is listed in parentheses.

b Calculated based on estimated NP densities and size: PrNC and PSA-PrNC, 0.9 g/mL; ENCP, 1.1 g/mL; PS-NP+ and PS-NP–, 0.2 g/mL (data from ThermoFisher).

c Based on fluorescent label. LOD varies between NP and experimental occasion but is typically 1 × 10^–8^ cm/s for NP *P*_app_.

d TAMRA-protamine *P*_app_ = 0.05 ± 0.05 × 10^–6^ cm/s.

e FITC-C12-R8 polymer *P*_app_ = 0.004 ± 0.003 × 10^–6^ cm/s.

f DiD label was undetectable by microscopy. Macroscopic evidence of strong mucus binding was seen.

g The permeating material of PrNC and PSA-PrNC in the Ussing chamber experiments was to some extent broken down as determined by dialysis of permeating material (see Figure S1). The permeability of intact PrNC and PSA-PrNC is approximately 50% lower.

h For complete data, see the supplement and Table S1.

i ND: Not determined. Values are given as average ± SD.

The permeability of the insulin cargo (see Table 2) was investigated using UPLC-MSMS. If the major part of the insulin incorporated into the permeated PrNC, PSA-PrNC, and ENCP (determined by fluorescence) had been transported intact, the final concentration of insulin in the acceptor chamber would have been well above the LOQ of the UPLC-MSMS detection method. However, in no instance could intact insulin be detected in the basolateral chambers. When the permeability of soluble insulin (used as a reference) was investigated in the dose interval spanning the dose range used in nanoparticle experiments (of 0.1–1 mg/mL), no insulin could be detected in the acceptor chamber. In summary, although three NP displayed measurable permeability across the human intestinal tissue *per se* as determined by fluorescent tagging, they were unable to deliver insulin to the acceptor chamber.

In Caco-2 monolayers, little permeability of the arginine-rich NP could be detected on the basolateral side, in most cases resulting in an NP *P*_app_ value of 0 based on NP fluorescence tags (data not shown). In previously published studies, the four arginine-rich NP showed higher permeabilities across Caco-2 monolayers than could be detected in the Ussing chamber and Caco-2 monolayer experiments reported here.^11−14^ Permeability of insulin across Caco-2 monolayers and into the receiving chamber was published for two of the NP (PARG NC and ENCP) while PrNC and PSA-PrNC did not deliver measurable insulin levels across the Caco-2 monolayers. Possible reasons for this discrepancy could be intralab variability in the experimental protocols, insulin assay reliability in the presence of high protease expression levels in Caco-2 cells, or possibly a result of different Caco-2 cell clones being used.^32^ For these reasons, relating NP permeability from Caco-2 monolayers to human jejunal tissues seems difficult. Whenever possible, permeability experiments should be prioritized for isolated intestinal tissues mounted in Ussing chambers that comprise all of the barriers to permeation.

Still, permeability studies across human tissue mounted in Ussing chambers also have some drawbacks. The tissue is compressed at the circumference of the chamber during tissue mounting, and can be damaged, resulting in artificial leakage of solutes and potentially NP.^33^ In our study, we used fluorescently labeled dextran with an average molecular weight of 4000 Da (FD4) to monitor this edge effect. In general, the edge effect was small, resulting in low *P*_app_ values for FD4 of <1 × 10^–8^ cm/s. Some tissues displayed an FD4 permeability >3 times higher than the average FD4 permeability. These tissues were classified as leaky and were excluded from further analysis (Figure S2F). Surprisingly, while FD4 exhibited increased permeability in such leaky tissues, no increase in NP permeability was observed, indicating that the NP were still too large to be influenced by the edge effect. In addition to the edge effect, an increased permeability has sometimes been observed at the location of small defects in the barrier, including apoptosis-derived single cell defects.^34^ Permeation via such defects is sometimes referred to as the unobstructed pathway and constitutes another low-capacity pathway that may contribute to a measurable but insignificant permeability of molecules or NP normally considered impermeable. *Ex vivo* experiments in Ussing chambers are also unable to fully capture the geometry and dynamics of the *in vivo* intestine.^33,35,36^

As the measured permeability of NP is based on fluorescence accumulation in the basolateral chamber, confirmation that the fluorescent markers were stably attached to the NP was explored. If the fluorophore is released, permeability will be overestimated compared to intact, labeled NP. To ascertain that permeability measurements were based on intact NP and not digested material, dialysis (3.5–5 kDa molecular weight cut off, MWCO) of samples from the receiving chambers and untreated NP was performed. None of the NP, prior to exposure to tissue in Ussing chambers, showed a significant reduction in fluorescence after dialysis, indicating that they retained their fluorescent dyes. However, dialyzed samples from the acceptor chamber at the end of the permeability experiments across jejunal mucosae displayed a different pattern. ENCP, PS-NP+, and PS-NP– all seemed to permeate the intestinal tissue in intact form as fluorescences of samples were unchanged after dialysis. PrNC and PSA-PrNC samples, however, showed a reduction in fluorescence after dialysis. This indicates a partial breakdown of these NP to fluorophore-containing fragments able to permeate the dialysis membrane (Figure S1). It is possible that released fluorescent label from PrNC and PSA-PrNC leads to overestimation of the permeability of these fluorophore-labeled NP (Table 2). Note that if the fraction of released fluorophore is disregarded (Figure S1), the permeability of the fluorescent NP becomes around 50% lower than the permeability value based on the total permeability of the fluorophore (Table 2).

Thus, while PrNC and PSA-PrNC were stable and protected their insulin cargo in the presence of 1% pancreatin, this stability could not entirely be extrapolated to the intestinal tissue with its large diversity of peptidases and proteases. It is therefore likely that the insulin cargo, along with the NP, is partially digested during passage over the jejunal epithelium. Thus, of the four investigated arginine-rich NPs, only ENCP permeated jejunal mucosa predominantly in intact form. It was concluded that future profiling of NPs intended for oral administration should include investigation of NP stability in intestinal tissue. To learn more about the intestinal barriers encountered by the four arginine-rich NPs, interactions with the jejunal tissue barriers were explored in more detail.

### NP Toxicity in Human Jejunum

All NP displayed low toxicity to jejunal tissue at concentrations of 0.2–5.8 mg/mL after 120 min exposure in Ussing chambers (Table 2, Table S1). No effects on tissue electrophysiology, viability, or ATP levels were observed during the Ussing chamber experiments. No significant stimulation of cytokine release from NP-exposed tissue could be detected (Table S2). This is not surprising, given that most NPs did not reach the epithelial cell layer in significant amounts (see below). However, ENCP exposure was associated with some release of LDH (Figure S3). This was probably a result of a stronger interaction between ENCP and the epithelial cell surface. For a full discussion of the negative toxicity results, see the Supporting Information.

### NP Mucus Binding and Permeation Across Human Jejunum

**Figure 3:**
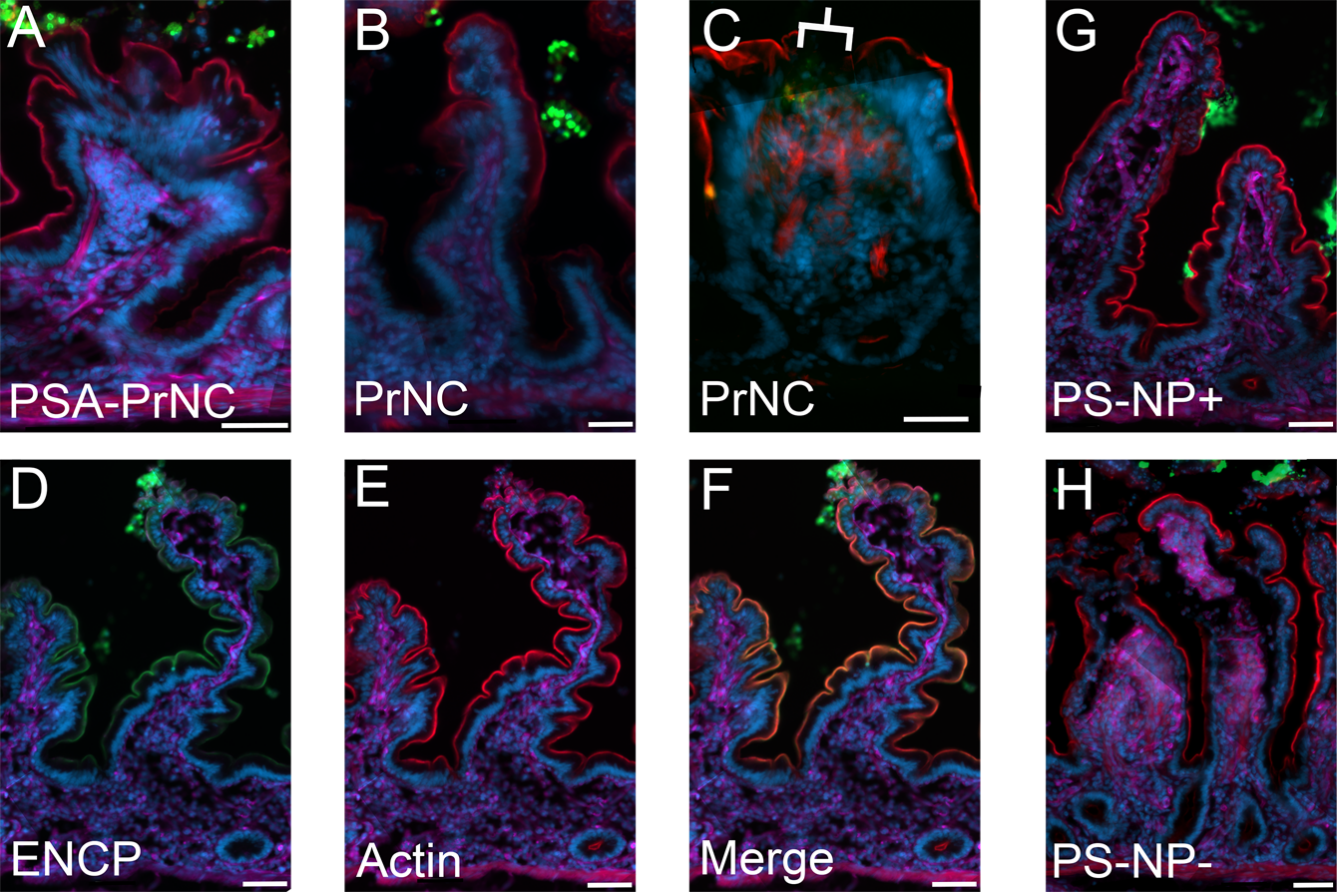
NP–tissue interactions in human jejunum. Images depict the distribution of NP in human jejunal mucosa with a mucus layer in Ussing chambers at 90 min after NP administration to the mucosal side. In all images, the NPs are stained green, actin is stained red, cell membranes are stained purple, and nuclei are stained cyan. The scale bar depicts 50 μm. (A) PSA-PrNC (green), (B) PrNC (green). NPs in panels A and B show pronounced mucus binding and little interaction with the epithelium. (C) PrNC (green) and PSA-PrNC (not shown) were in rare cases found below the epithelial surface. At these sites, the epithelial cell layer was missing, and hence, the epithelial barrier was defective as indicated by the lack of actin staining of the apical BBM (red). The defect is indicated by a white bracket. One single villus is shown. Figure S7 shows another example of PrNC localized in the lamina propria below an epithelial defect. (D) ENCP (green) showed little to no interaction with the mucus but was found at the apical BBM of jejunal enterocytes, creating a green outline of the epithelial surface (see Figure 4 for higher resolution). (E) Actin staining outlines the brush-border membrane in red. (F) Nearly total colocalization of the ENCP signal (green) and brush-border membrane actin stain in red, resulting in an orange overlay indicating ENCP localization to the BBM. (G) PS-NP+ (green) and (H) PS-NP– (green); both polystyrene control NP, PS-NP+ and PS-NP–, showed pronounced mucus binding and little to no interaction with the epithelium. All NP were imaged in at least five sections in tissue specimens derived from at least two parallel Ussing chambers from each of two donors.

In contrast to the Caco-2 monolayer model, human jejunal tissue retains an intact mucus layer in the Ussing chambers.^36,37^ It was therefore possible to investigate to what extent different NP permeated the mucus layer by microscopy. In all cases NP were studied in at least two Ussing chamber experiments on separate days with tissue from two different donors. From each of these experiments five or more microscopy sections from at least two tissue specimens in parallel Ussing chambers were imaged. Tissue was exposed to NP for 90 min. PrNC and PSA-PrNC as well as PS-NP+ and PS-NP– all showed pronounced mucus binding and were to a large extent trapped in the mucus layer on the apical side of the intestinal mucosae when mounted in chambers (Figure 3). The fluorescent label of PrNC and PSA-PrNC, TAMRA-protamine, did not show any mucus binding (Figure S5). In previous *in vitro* studies using purified porcine mucin and cocultures of Caco-2/HT29-MTX cells, PSA-PrNC showed lower mucus binding than did PrNC.^12^ Despite this, there was no improvement of the mucus penetration of PSA-PrNC as compared to PrNC in Ussing chamber experiments with human jejunum. These results underscore the importance of studying mucus penetration in native mucus with an intact 3D structure. The possibility to improve the mucus barrier function of *in vitro* model systems has been reviewed previously.^38^

PrNC and PSA-PrNC were occasionally found in small areas beneath the epithelium (Figure 3C, Figure S7). In all cases this occurred in close vicinity to small defects in the epithelial cell layer (see below). PrNC and PSA-PrNC were also found to bind enterocytes that had been shed into the mucus layer (Figure 3A,B). Free TAMRA-protamine could not be visualized in the tissue on any occasion (Figure S5).

**Figure 4:**
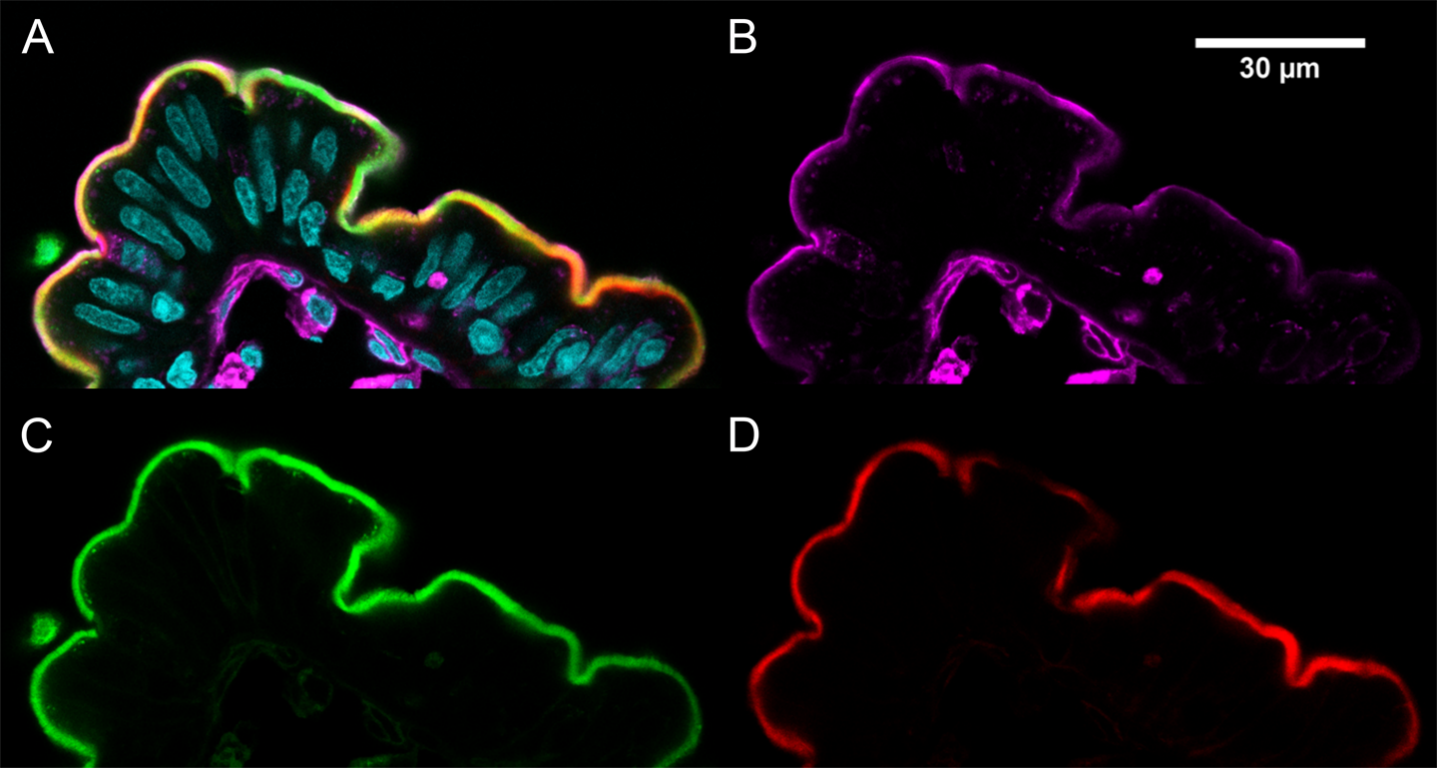
Localization of ENCP in enterocyte brush-border membranes. The LSM image shows that the vast majority of endocytosed ENCPs are localized in the enterocyte brush-border membrane after 90 min of exposure in the Ussing chamber. (A) Overlay of all channels. Nuclei stained with DAPI. (B) Membrane staining in purple by wheat germ agglutinin. (C) ENCPs in green are localized to the brush-border membranes (BBM). Some endocytosed nanoparticles are seen in punctate stain just beneath the epithelial BBM. (D) Red phalloidin staining of actin visualizing the BBM. ENCP was imaged in several sections in tissue specimens from two parallel Ussing chambers from two donors.

ENCP on the other hand did not bind to the jejunal mucus layer, and a significant portion of the ENCPs permeated the mucus layer and reached the jejunal epithelium where the NPs were found in close proximity to the brush-border membrane (BBM) (Figures 3 and 4). This is in agreement with the permeability experiments, showing that ENCP permeated the jejunal tissue in intact form. The final NP, PARG NC, could not be detected by microscopy in jejunal samples in Ussing chambers. PARG NC seems to a large extent to be bound to the superficial loosely adherent mucus layer which might be sloughed during washing and processing of samples. The fluorescent label of this NP is dissolved in an inner oil phase and not covalently attached to the NP, making it possible that the label is washed away during sample processing.

**Figure 5:**
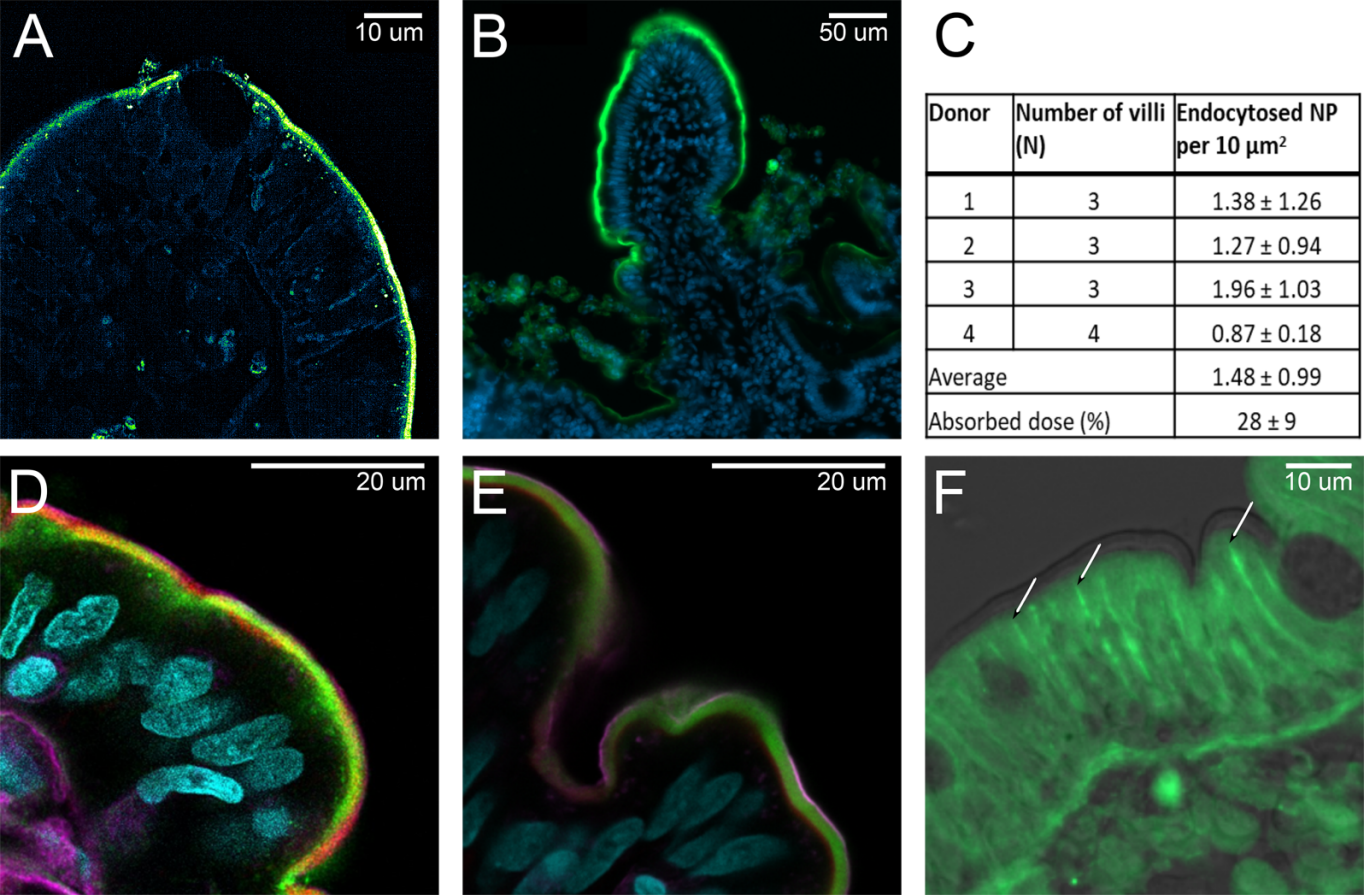
Quantitation of internalized ENCP in jejunal epithelial enterocytes. (A) SIM imaging allows quantitation of ENCPs in enterocytes. When added to the apical side of jejunum, ENCP were primarily found in the BBM with few particles in the basal regions of the cells and nearly no NPs in the subepithelium. All enterocytes internalized ENCPs, but no NPs were detected in goblet cells (black lacuna). ENCP labeled in green. (B) Approximately 75% of the villi surface had absorbed ENCP (green) after 90 min exposure. Actin staining was omitted for clarity. (C) Quantitation of the number of ENCP absorbed per unit area in villi in tissues from four donors. Values are given as average ± SD. (D) ENCP (green) were, after apical addition, localized to the BBM and were internalized into punctate stains reminiscent of early endosomes. (E) Free FITC-C12-R8 polymer (green) stained the BBM but did not enter endosomes. (F) When added to the basolateral chamber ENCP (green) diffused unhindered through the lamina propria to the epithelial tight junctions at the upper basolateral edge of enterocytes (arrows). No ENCP were visualized within the BBM. In all LSM images (B–F), ENCP or FITC-C12-R8 are stained green, actin is stained red, and nuclei are stained cyan; in addition, in panels D and E, cell membranes are stained purple. All sections were processed after 90 min in the Ussing chambers.

To understand NP binding to mucus and human jejunal tissue, the amount of fluorescence remaining in the apical donor chamber was measured at the start and end of the experiments (Figure S6). It was apparent that PS-NP+, PS-NP–, PARG NC, PrNC, and PSA-PrNC showed a pronounced binding of 60–99%. ENCP displayed a binding of approximately 40%. Free TAMRA-protamine, FITC-C12-R8, and FD4 (hydrophilic control) showed little binding to mucus and tissues. For ENCP the uptake into the enterocytes was approximately 30% (see Figure 5), and the remaining mucus binding then becomes approximately 10% (Figure 3). Other NP showed little interaction with the epithelium, and the detected binding is most probably binding in the mucus layer (Figure 3). The mucus binding of the NP is reminiscent of *in vivo* defenses against foreign “particles” such as bacteria and viruses.^39,40^

NPs interact with mucus via electrostatic interactions, steric hindrance, and hydrophobic interactions.^16^ Much focus has been on ζ-potential where a positive charge is believed to promote mucus binding via electrostatic attraction. However, several studies indicate that it is the magnitude of the ζ-potential, rather than the sign, that drives mucus binding.^16,25,41^ This is in accordance with our results, where the only mucus-penetrating NP, ENCP, had a near neutral ζ-potential (Tables 1 and 2). NPs displaying large positive or negative ζ-potentials showed significant mucus binding (PARG NC, PS-NP+, PS-NP−). Importantly, other molecular interactions are also at play. It is likely that the strong mucus binding of PrNC and PSA-PrNC, both exhibiting near neutral ζ-potentials, is due to hydrophobic interactions and/or sterical hindrance due to their larger size (>300 nm, Table 1). Another possibility is that cationic domains on the NP surface are formed by aggregations of polyarginine or protamine molecules, and that these cationic areas bind to the mucus mesh.

Studies with mucus-inert NP and virus particles show that their diffusion in intact mucus starts to slow down at a particle diameter above approximately 100 nm and that diffusion declines very rapidly at particle sizes above 200 nm.^42^ An analysis of the pore diameter in porcine ileal mucus showed that 90% of the pores in the mucus mesh lie within 100–300 nm in diameter.^43^ If the pore size for human GI mucus is comparable to that of the porcine mucus, NP larger than 300 nm, such as PrNC and PSA-PrNC, should experience severe steric hindrance in the mucus mesh.

ENCPs had a smaller diameter of approximately 200 nm. They were designed to be mucus permeating in that a dense cover of short PEG-chains gave these NP a hydrophilic and nearly net neutral surface mimicking mucus diffusive properties of virus capsids.^44^ These surface properties have previously been shown to minimize interactions with the mucus mesh and allow mucus penetration.^16,25,41,44^

### Transepithelial NP Transport in Human Jejunum

PrNC and PSA-PrNC fluorescence was only partly released during the transepithelial transport, and their localization above and beneath the epithelial barrier could therefore be investigated by microscopy (Figure 3A,B). In all cases, subepithelial PrNC and PSA-PrNC were found close to small defects in the epithelial barrier (Figure 3C). These epithelial defects, defined as loss of small sections of the epithelial layer of enterocytes, were found in on average every tenth villus and were not observed after exposure to the other NP, indicating that the epithelial defects were a result of the PrNC and PSA-PrNC exposure. In accordance with the possible induction of epithelial damage and a leak pathway, FD4 permeability showed a trend toward higher values and showed a significantly larger variability in permeability in PrNC and PSA-PrNC-exposed tissues (Figure S2H). These NP showed no toxic effects on the tissue in other experiments, and it is possible that the epithelial defects are due to an experimental artifact. In Ussing chamber experiments, the lack of circulation and resulting water accumulation in the tissue can lead to bubbles of epithelium swelling on the tips of the villi.^45^ These bubbles are removed before microscopy by sucrose treatment of tissue samples during fixation, to avoid sectioning damage to the villi. If PrNC and PSA-PrNC destabilize the tissue bubbles during the experiments, this could lead to the observed epithelial defects (Figure 3C and Figure S7).

Fluorophore signals from ENCP suggested that there was internalization of the NP by jejunal enterocytes colocalized with the BBM (Figures 3D–F and 4). The signal from ENCP also suggested uptake in the epithelial layer. The ENCPs were evenly absorbed over the upper part of every villus, staining the brush-border membrane of every enterocyte while goblet cells were devoid of absorbed ENCP (Figure 5A). Below the brush-border membrane, discrete fluorescent spots could be detected, reminiscent of NP absorption into endosomal structures (Figures 3F and 4). The basolateral pole of the enterocytes and the subepithelial portions of the villi were devoid of ENCPs (Figures 3F and 4).

The epithelial associations of the NPs in isolated human jejunal tissues were in qualitative agreement with studies in the Caco-2 model (see Figure S4). PARG NC could barely be detected in Caco-2 cells.^11^ PrNC and PSA-PrNC were found associated to the apical Caco-2 membrane, but little staining was seen within the epithelial cell layer.^12,14^ ENCP, on the other hand, were more strongly associated with the Caco-2 cells and were taken up by the epithelial cells by endocytosis.^13^

ENCP combines two distinct and partially opposing features that favor access to and uptake by the intestinal epithelium *ex vivo*: first, a surface layer of PGA–PEG allowing unhindered diffusion through the mucus and, second, a core containing the CPP R8 that, when exposed, can interact with the plasma membrane and stimulate epithelial uptake. ENCP had a smaller diameter than the other NP, a factor possibly contributing to penetration of both the mucus layer and apical membrane of enterocytes (Table 1). In previous studies, an uncoated version of ENCPs called NCP showed higher Caco-2 uptake than what was seen after addition of the PGA–PEG coat to form ENCP.^13^ The reduced uptake of ENCP was presumably due to a partial shielding of the R8 moieties that were exposed in the NCP. NCP displayed a strong positive ζ-potential, and thus strong mucus binding, and would have been unsuitable for administration to mucus-covered tissue. It was therefore not included in this study. As the PGA–PEG coat of ENCPs is not covalently attached, it is possible that this coat is partially shed during or after passage through the mucus layer, exposing more R8. Such a strategy was used for CPP-containing NPs with an *N*-(2-hydroxypropyl) methacrylamide copolymer coat that was partially shed during mucus penetration allowing mucus diffusion combined with efficient epithelial NP uptake.^20^

In conclusion, NPs could be classified into three groups according to their stability and interaction with the jejunal epithelium: (1) Mucus binding resulting in very low permeability (PARG NC, PS-NP+, and PS-NP−); (2) mucus binding but with measurable permeability and epithelial uptake at spots close to tissue defects possibly caused by the NP (PrNC and PSA-PrNC); and (3) mucus-penetrating NPs associated with significant uptake into, and some transport across, the epithelial barrier (ENCP).

### Quantitation of ENCP Absorption

The jejunal villous epithelium internalized ENCP in comparatively large amounts (Figures 3–5). This allowed quantification of the number of internalized ENCP by human jejunal tissue in Ussing chambers by the aid of structured illumination microscopy (SIM) (Figure 5A). Using SIM individual ENCPs could be visualized in the BBM and epithelial cell layer allowing quantitation of internalized NPs. Measuring the length of the villus surface together with the known thickness of the microscopy sections allowed the exposed villus surface area to be estimated and the absorbed ENCP per unit area to be calculated. Figure 5B depicts the surface of the jejunal villi that had absorbed ENCPs after a 90 min incubation in the Ussing chamber. Enterocytes covering 75% of villus surface endocytosed the ENCPs. This is in agreement with the hypothesis that the “absorptive surface area” in the intestine increases with reduced permeability of the permeating molecule.^46,47^ A highly permeable compound is rapidly taken up and interacts mostly with the villi tips before being removed from the lumen. Low-permeable compounds or NPs will remain longer in the intestinal lumen and therefore have more time to diffuse down between villi prior to internalization. They will thus be able to access a larger epithelial surface area than highly permeable compounds. Few or no ENCPs were found in the jejunal crypts.

The number of ENCPs internalized per unit area after apical-side addition was comparable between samples from separate human donors, averaging 1.48 ± 0.99 ENCPs per 10 μm^2^ of epithelial surface (Figure 5C). Scaling this number to the jejunal surface area exposed in the Ussing chamber, including surface amplification by villi (scaling factors taken from Helander et al., 2014)^48^ and the absorptive portion of the villi (visualized in Figure 5B), allowed estimation of the number of ENCPs taken up by exposed intestinal tissue (see the Methods section). Based on this area the number of ENCP absorbed per Ussing chamber compared to the total amount of ENCP (1.5 mg/chamber) given in the experiment amounted to 28 ± 9% (Table 2). By comparison, in studies on ENCP uptake by Caco-2 cells, 48 ± 6% of added ENCP (0.2 mg/mL ENCP, corresponding to the lowest concentration used in Ussing chamber experiments) were taken up, and 2% were transcytosed by the Caco-2 cells.^11^ In contrast, only 0.2% of the given dose of ENCPs permeated across the jejunal tissue into the acceptor chamber of the Ussing chamber (Table 2). Extrapolating the Ussing chamber *P*_app_ values for ENCPs in our PBPK model (incorporating jejunal surface area, volume, and transit time) yields a fraction absorbed *F*_a_ = 0.3% (Table 2).^32^ However, this PBPK model assumes the absorptive surface of the jejunum equates to a smooth tube. Extending the absorptive surface to 75% of the villi surface increase the predicted *F*_a_ to 1.93% (see the Methods section).

While a significant amount of ENCPs were taken up by the jejunal enterocytes, very few of the particles were subsequently transported across the basolateral membrane into the lamina propria (Figure 5A,D). The majority of endocytosed ENCPs remained inside the cells, predominantly in the apical pole above the terminal web (Figure 5A,D). Microvilli of the human small intestine show a typical diameter of approximately 100 nm.^1^ This is less than half the diameter of the ENCP and suggest that these NPs might be trapped in the BBM. Another possibility is that the cytoskeletal terminal web supporting the brush border at the apical pole of the enterocytes impedes NP movement further into the cell. The pore size of the terminal web has been estimated to 100 nm, and the web could impede the movement of larger particles.^49,50^

We next investigated if the free polymer, C12-R8, constituting the backbone of the ENCP could pass the microvillus and terminal web barriers. Figure 5D,E compares the internalization of ENCPs (Figure 5D) with the same concentration of free FITC-C12-R8 polymer in solution (Figure 5E). ENCP gave rise to a punctate staining pattern below the BBM that is reminiscent of apically located early endosomes (Figure 5D). In contrast, free FITC-C12-R8 polymer strongly stained the BBM but did not seem to be internalized into endosomes (Figure 5E). This is consistent with the *P*_app_ value for ENCP being 100-fold higher than the *P*_app_ for the free FITC-C12-R8 polymer (Table 2). It is established that R8 destabilizes membranes and induces endocytosis once it reaches a sufficient concentration on the membrane. Thus, the incorporation of R8 into NP seems to ensure a sufficiently high local peptide concentration to allow membrane penetration and endocytosis. It has also been demonstrated that R8 binds RNA and accumulates in the nucleolus.^51^ FITC-R8 can be used to stain this organelle. In our experiments with ENCPs and FITC-C12-R8 polymer, however, no staining of the nucleolus could be detected, suggesting that little of the labeled R8 entered the cytoplasm of jejunal enterocytes.

The subepithelial lamina propria did not present a major barrier to ENCP diffusion in the basolateral to apical direction (Figure 5F). ENCPs added to the basolateral chamber diffused through the lamina propria to the epithelial tight junctions at the upper basolateral edge of the enterocytes. No ENCPs were found within the BBM after basolateral addition. This implies that once ENCPs added from the lumen side have passed the rate-limiting mucus and epithelial barriers, they will diffuse without much hindrance through the submucosa to reach the capillary bed. To achieve systemic insulin delivery, insulin needs to be released from ENCP in the submucosa after passage across the epithelium. The ENCP are too large to enter the capillary pores, which have an average diameter of less than 10 nm, so the insulin needs to be released before the ENCPs reach that barrier. Alternatively, it could be speculated that intact NPs could be transported away from the intestine via the lacteals and lymphatic system as are chylomicrons. Further investigation of this alternative NP pathway was outside the scope of this investigation.

### Mechanism of ENCP Permeability

**Figure 6:**
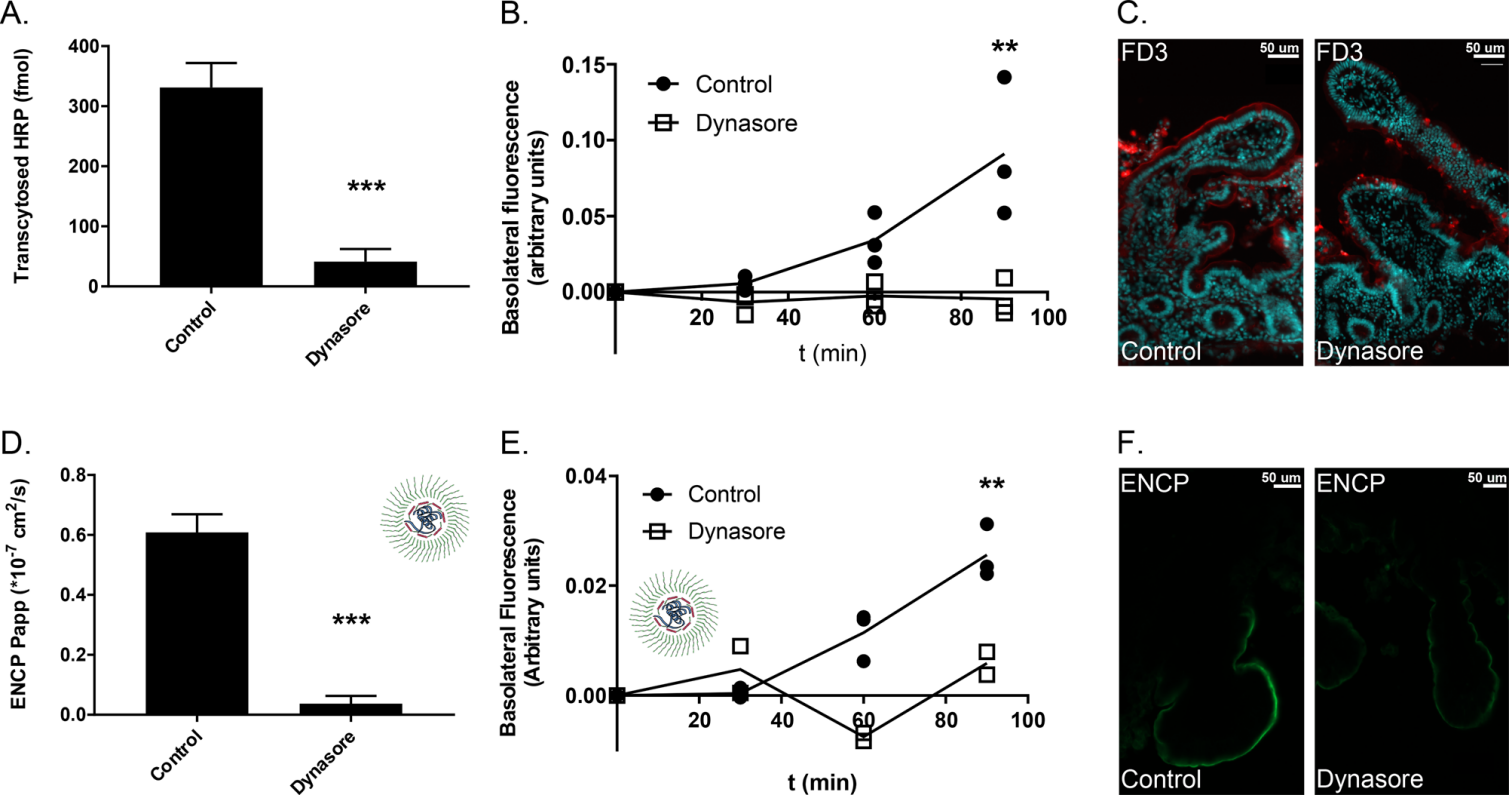
ENCP permeability mechanism across human jejunal mucosa. (A) Permeability of HRP in the presence and absence of the dynasore. (B) Dynasore reduced permeability of FD3. (C) Reduced endocytosis of FD3 in the presence of dynasore illustrated by confocal microscopy. FD3 in red; nuclei stained in cyan. (D, E) Dynasore effects on ENCP *P*_app_ and permeability. (F) Dynasore seems to reduce internalization of ENCPs into enterocytes. ENCP in green. Images from control and dynasore treated tissue specimens were imagined and processed using identical settings. Average ± SD is shown, *n* = 3, ** *p* < 0.01, *** *p* < 0.001.

The epithelial uptake mechanism of ENCP in the human jejunal epithelium was investigated using the dynamin inhibitor, dynasore.^52^ Dynamin is instrumental in internalization of endosomes and transport of endosomes along the microtubule network, and its inhibition slows down or stops most endocytotic and transcytotic processes. In control experiments, dynasore pretreatment prevented endocytosis of lysine-fixable FITC-dextran 3000 (FD3) and reduced transcytosis of the marker horseradish peroxidase (HRP) (Figure 6A–C). Dynasore also inhibited ENCP transepithelial permeability as measured by fluorescence of labeled NP (Figure 6D–F). The ENCP *P*_app_ was reduced by 90% after dynamin inhibition (Figure 6D,E). Dynasore treatment seemed to reduce endocytosis of ENCPs into enterocytes, diminishing the ENCP signal in the BBM (Figure 6F). This indicates that ENCP entry into enterocytes is dependent on endocytosis and that dynamin-dependent transcytosis mediates most ENCP permeability. In contrast, dynasore did not influence PrNC permeability in Ussing chambers (Figure S8). As PrNC showed very little interaction with the epithelium (Figure 3), its permeability is unlikely to be influenced by dynamin activity.

A major drawback when studying endocytosis mechanisms is the lack of specificity of various chemical inhibitors of different endocytotic mechanisms.^53^ While chlorpromazine and filipin are considered relatively selective inhibitors of clathrin- and caveolin-dependent endocytosis, respectively, they display some cross reactivity. For macropinocytosis and phagocytosis, no selective inhibitors currently exist, making conclusive identification of endocytotic pathways difficult. The picture is further complicated by several clathrin- and caveolin-independent endocytotic mechanisms that are different form the classical endocytotic pathways.^54^ Considering these uncertainties, dynasore was chosen as a pan-endocytosis inhibitor. Contribution of individual endocytosis pathways was not studied further. In previous studies in Caco-2 cells, endocytosis of PrNC, PSA-PrNC, and ENCPs was partially inhibited by chlorpromazine and filipin, suggesting involvement of multiple endocytotic pathways in this cell line.^11−14^

Jejunal enterocytes are considered to show low endocytotic activity. In contrast, this study found endocytotic capacity of the jejunal epithelium to be significant. It is also striking that endocytosis is the most strictly regulated process in enterocytes, at the protein level, indicating the importance of this process for the physiological function of the intestinal epithelium.^55^ Of the top 100 most tightly regulated proteins in the jejunal mucosa, 45 are involved in endocytosis and intracellular vesicular transport. However, the observed endocytosis of ENCPs was not sufficient to support transepithelial permeability. The most common pathways for endocytosed material is either recycling to the cell membrane of origin or transport to lysosomes. In lysosomes, both the peptidic ENCP and the insulin cargo would be at risk of digestion, thereby diminishing any chance of transcytosis of either. It is possible that permeability of ENCP, or other endocytosed NP, could be increased by adding a more effective endosomal escape functionality to the NP that could facilitate escape into the cytosol. Such strategies have previously been attempted for other NP with some success.^56^

### *In Situ* Administration of NP in Rat Jejunal Loops

The final goal of NP formulation of biologics is the possibility of their successful oral administration. In view of the *ex vivo* results the expected *in vivo* delivery capacity of these NP is likely to be limited. However, *in vitro* experiments with Caco-2 monolayers were not in agreement with the isolated jejunal tissue studies and showed larger permeation of some of the NP.^12,13^ Insulin-containing NP were therefore administered to rat intact jejunal loops *in situ*, and the resulting pharmacokinetics and pharmacodynamics were monitored.

**Figure 7:**
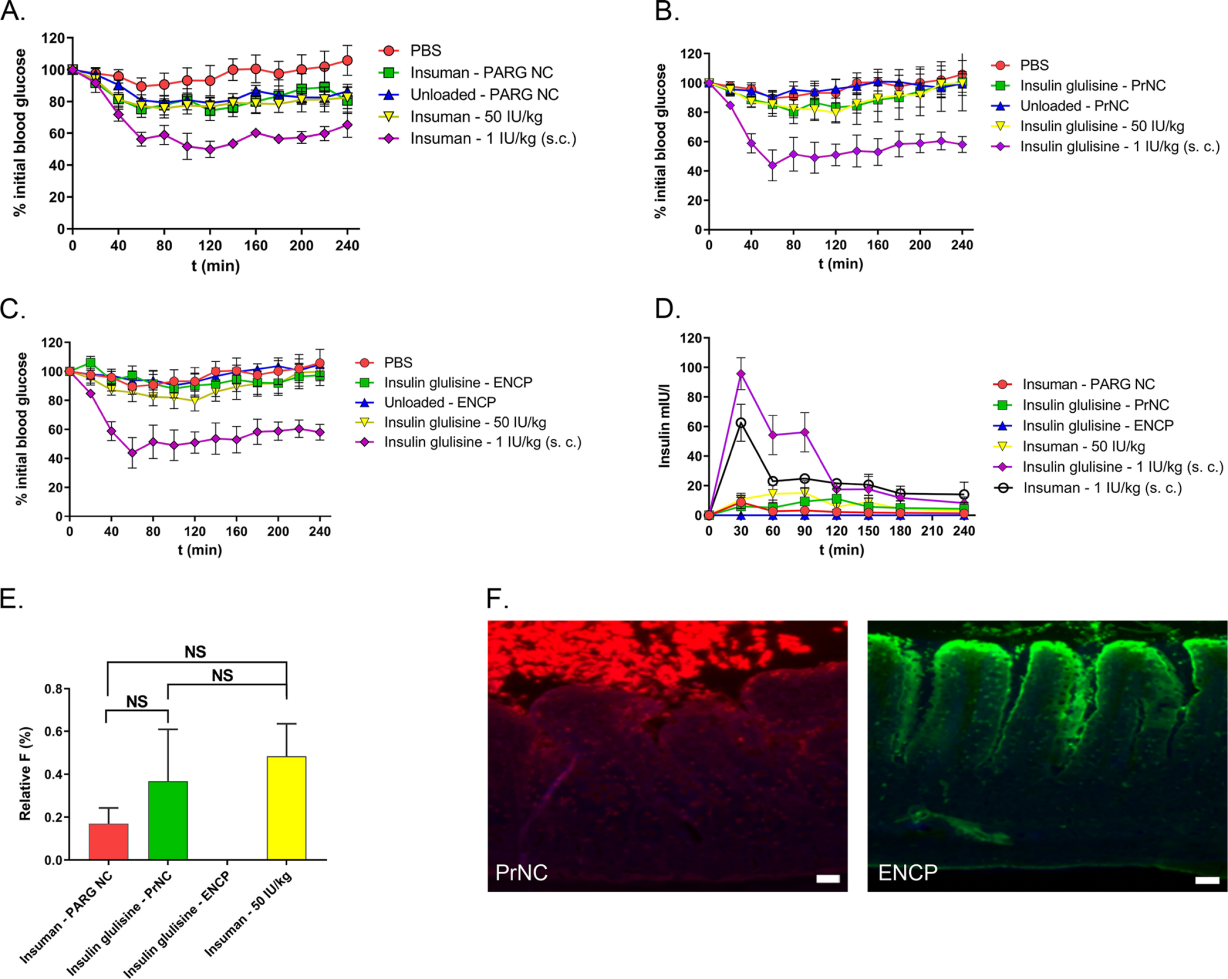
*In vivo* administration of insulin-loaded NPs to rat jejunal loops. Pharmacodynamics of blood glucose responses: (A) PARG NC; (B) PrNC; and (C) ENCPs. (D) Concentrations of human insulin in blood in dosed rats. (E) Relative bioavailabilities for NP administered to rat jejunal loops compared to sc administration (*F*_sc_ = 100%). (F) Confocal microscopy of fluorescent NP interactions with the rat jejunal mucosa. PrNC in red. ENCP in green. Scale bar equals 50 μm. Average ± SEM is shown, NS = not significant.

No statistically significant effect on blood glucose was seen after instillation of PARG NC, PrNC, or ENCPs carrying 50 IU/kg either human insulin or glulisine into rat jejunal loops (Figure 7A–C), and neither was the area above the blood glucose concentration–time curve (AAC) significantly different from controls after jejunal administration of NP or free insulin (Figure S9). Insulin administered sc to rats induced a statistically significant increase of the AAC. As PrNC and PSA-PrNC had shown very similar results in previous rat *in vivo* experiments using oral gavage, PrNC alone was selected for instillation in the jejunal loops.^12,14^ Administration of 50 IU Insuman solution in the jejunal loop did not reduce blood glucose levels. In contrast, after sc injection of 1 IU/kg Insuman rats responded with a large drop in blood glucose levels (Figure 7A–C). After administration of PARG NC, PrNC, or 50 IU free Insuman to the intestinal loops, a rise in circulating human insulin could be detected, indicating low-level absorption or leakage of the peptide into circulation (Figure 7D). The bioavailability of NP delivered insulin was 0.3 ± 0.2% for PrNC and PARG NC formulations, and 0% for ENCP (Figure 7E), in no case significantly different from insulin solution. This suggests that the low amounts of human insulin detected in the circulation mainly derive from leakage through small defects in the gut wall, either naturally occurring or introduced by the experimental procedure. Figure S10 shows that the intestinal loop showed normal anatomy without distension after NP administration, along with the low levels of fluorescence background seen in tissue sections.

In general, healthy rats are less sensitive to changes in plasma glucose than diabetic rats, and the former show less variability in their glucose levels and insulin responses, minimizing the risk for false positive and negative results.^57^ The plasma glucose concentration is a sensitive parameter that can be affected by stress and handling rats, as well as by anesthesia.^57^ PK monitoring of human insulin in the blood alleviates many of these sources of variability and gives a more robust result than plasma glucose PD, increasing the certainty that the tested NP did not enhance the delivery of insulin across rat jejunum beyond 1% relative bioavailability.

In previous studies, these NPs have been administered to healthy, conscious rats by intraduodenal (PARG NC), intrajejunal (PrNC and PSA-PrNC), and oral administration in enteric capsules (ENCPs).^11−14^ In these studies administration of PARG NC led to small reductions in plasma glucose levels while no effects of ENCPs on rat plasma glucose were observed. After intrajejunal administration of PrNC and PSA-PrNC, statistically significant reductions of blood glucose by approximately 30% were observed from 1 to 3 h after administration. It is difficult to compare these different administration routes, and while reductions in plasma glucose were detected in previous studies, measurements of plasma insulin were not performed. This leaves open the question what bioavailability was achieved for insulin in those studies. Rat jejunal loops contain high levels of mucus as released mucus cannot be transported away from the loop. Such a reinforcement of the mucus barrier in intestinal loops could be an explanation for the lower blood glucose responses seen after PrNC instillation to jejunal loops compared to intrajejunal administration in previous studies. Isoflurane anesthesia has also been shown to diminish jejunal motility, an effect that could possibly lead to lower interactions between NPs and the intestinal epithelium in a sedated animal.^58^

Microscopic imaging of NP interactions with the rat jejunum following instillation verified our *ex vivo* findings in human jejunum and was in agreement with previous *in vitro* findings (Figure 7F). PARG NC could not be detected by microscopy, possibly due to a loss of the dissolved DiD label during sample processing. PrNC showed a pronounced mucus binding and little interaction with the epithelium. ENCPs on the other hand did not bind to mucus but were found below the mucus layer at the level of the jejunal epithelium covering the top and flanks of the villi. NP distribution patterns *in situ* in rat jejunum were consistent with those observed in isolated human jejunum but not with observations in Caco-2 monolayers, again underscoring the importance of the mucus barrier. The rat *in situ* data is in good agreement with our Ussing data with regard to both NP distribution and lack of pharmacologically relevant permeability over the intestinal wall. After oral administration *in vivo* the NP will also be subjected to the harsh environment of the stomach. By using enteric coated capsules for the NP, this barrier can be circumvented in larger animals. However, the small size of capsules suitable for rodents results in limited loading capacity and poor prediction of performance in larger species, making jejunal administration of NP suspensions the initial screening method of choice in small animal models.^59^

## Conclusions

While no NP resulted in efficient delivery of insulin across human or rat jejunal epithelium *ex vivo* or *in situ*, efficient delivery of ENCPs into the intestinal epithelium was discovered. By using human jejunum in Ussing chambers, NP interactions with the major intestinal barriers to absorption could be investigated in detail, and these experiments were more predictive of *in vivo* results than *in vitro* studies in the Caco-2 cell model. This could reduce the risk of attrition when NP are transferred from *in vitro* to *in vivo* testing during development of delivery systems for oral biologics. The main barriers to intestinal permeability of NP were the mucus layer and the intestinal epithelium. The mucus barrier impeded both positively and negatively charged NP but could be overcome when the surface had a net neutral charge and a high hydrophilicity.

While the CPP R8 facilitated NP endocytosis into enterocytes, it was not sufficient to induce transport across the epithelium. Further optimization by incorporation of a more efficient endosomal escape function than R8 could possibly increase cytosolic exposure and, if transcytosis follows, oral bioavailability of therapeutic cargoes.

The mucus binding of PARG NC, PrNC, and PSA-PrNC is likely to limit their usefulness for local or systemic drug delivery following oral administration. Strong mucus binding will lead to interactions with the loosely attached mucus layer resulting in rapid release from the intestinal wall into the lumen. Nevertheless insulin-loaded PrNC and PSA-PrNC have previously been found to lower rat blood glucose levels after administration to the GI tract.^12,14^ The mucus-penetrating and highly endocytosed ENCP shows promise for oral drug delivery of small molecule drugs or biologics to the intestinal mucosa. Although ineffective for oral delivery of insulin, ENCP permeability through the various intestinal barriers suggests that this NP shows potential for local delivery in treatment of, e.g., inflammatory bowel disease or colorectal cancer.

## Methods

### NP Preparation and Characterization

#### Materials for NP Preparation

Sanofi (Paris, France) kindly provided recombinant human insulin analogues (Insulin glulisine, *M*_w_ 5823 Da; Insuman *M*_w_ 5808 Da). Professor Ernest Giralt (Institute for Research in Biomedicine, Barcelona, Spain) kindly provided lauric acid–octarginine (C12-R8) and FITC labeled-C12-R8 (FITC-C12-R8). Diblock {m[PEG]_455_-*b*-[PGA]_10_, methoxy-poly(ethylene glycol)-block-poly(l-glutamic acid sodium salt), *M*_w_ = 22 kDa, 20 kDa PEG, and 2 kDa PGA} was purchased from Alamanda Polymers (Huntsville, AL). Poly-l-arginine (*M*_w_ 26–37 kDa) was purchased from Polypeptide Therapeutic Solutions (PTS, Valencia, Spain). Pharmaceutical grade poloxamer 188 was purchased from BASF (Ludwigshafen, Germany). Pharmaceutical grade oleic acid and Span 80 were purchased from Croda (Snaith, UK). Pharmaceutical grade sodium deoxycholate (SDC) was purchased from New Zealand Pharmaceuticals (Palmerston North, New Zealand). The 1,10-dioctadecyl-3,3,30,30- tetramethylindodicarbocyanine perchlorate fluorescent dye (DiD oil) was obtained from Life Technologies (Eugene, OR).

Protamine sulfate of low *M*_w_ (5 kDa, derived from salmon) was purchased from Yuki Gosei Kogyo, Ltd. (Tokyo, Japan). The stabilizing surfactants, polyoxyethylene 40 monostearate (PEG-st 40), Croda Europe Ltd. (Snaith, UK), and sodium glycocholate (SGC) from Dextra (Reading, UK), were obtained. Caprylic/capric triglyceride (Miglyol 812) was from Cremer, Oleo Division, (Witten, Germany). Colominic acid sodium salt (polysialic acid, PSA) was from Nacalai tesque INC, (Tokyo, Japan). 5-TAMRA, SE (5-carboxytetramethylrhodamine, succinimidylester, single isomer), was purchased from Emp Biotech (Berlin, Germany). Ultrapurified water was obtained from a Millipore Milli-Q Plus water purification system (Darmstadt, Germany). All other chemicals were of analytical grade.

#### Preparation of Polyarginine (PARG)–Oleic Acid–Insulin Nanocapsules (PARG NC)

PARG NC were prepared by a modified solvent displacement technique.^60^ Insulin (Insuman) was dissolved in 0.01 N HCl (pH ∼2.1) at a concentration of 15 mg/mL, and 0.1 mL of this solution was transferred to an organic phase composed of 62.5 μL of oleic acid, 20 mg of Span80, 2.5 mg of SDC, 4.1 mL of acetone, and 0.8 mL of ethanol. This organic phase was mixed using a vortex agitator (VELP Scientifica, Usmate, Italy) and immediately transferred into 10 mL of 30 mM pH 5.5 acetate buffer, which contained 0.05% (w/v) PARG and 0.25% (w/v) poloxamer 188. After magnetic stirring for 10 min, the solvents were evaporated under a vacuum, decreasing the volume of the final formulation from 15 to 5 mL in a Rotavapor instrument (Heidolph Hei-VAP Advantage, Schwabach, Germany). Fluorescent NCs were produced by adding 50 μg of DiD to the organic phase instead of incorporating insulin. The absence of dye leakage was assessed upon incubation of the NCs in PBS and cell culture media, at 37 °C for up to 4 h.

#### Preparation of Insulin-Loaded Protamine Nanocapsules (PrNC)

Insulin-loaded protamine nanocapsules (PrNC) were prepared by the solvent displacement technique previously described.^12,60^ Briefly, PEGstearate-40 (16 mg), sodium glycocholate (5 mg), and Miglyol812 (59 mg) were dissolved in 3 mL of ethanol. Acetone (1.95 mL) was then added to this lipid phase followed by the addition of 1 mg of insulin (Insulin glulisine) dissolved in 50 μL of 0.01 M HCl. This organic phase was immediately poured over 10 mL of an aqueous phase containing 0.15% w/v protamine under magnetic stirring at 300 rpm. The molar ratio of insulin to protamine was 1:8. The elimination of organic solvents was performed by evaporation under a vacuum (Rotavapor Heidolph, Germany) to a final volume of 5 mL. Finally the nanoparticles were isolated by ultracentrifugation (Avanti J-E, Ultracentrifuge, Beckman Coulter) at 80 000*g* for 1 h at 15 °C.

Nanocapsules with a double protamine/polysialic acid (PSA) polymer layer were obtained upon addition of 0.1 mL of PSA solution (concentration: 3 mg/mL) to a volume of 0.5 mL of NCs (concentration: 18.6 mg/mL) under mild shaking at 300 rpm for up to half an hour generating PSA-covered PrNC (PSA-PrNC). The final protamine/PSA ratio was 5:1 (w/w).

For tissue uptake studies, fluorescent PrNC were prepared with TAMRA-labeled protamine (TAMRA-protamine). Protamine (10 mg) was dissolved in 0.1 M sodium bicarbonate buffer (1 mL, pH 8.60), and TAMRA (10 mg/mL in DMSO) was slowly added under mild stirring (300 rpm). After 1 h of incubation with mild stirring at room temperature, the labeled protamine was dialyzed for 72 h to remove free TAMRA (SnakeSkin, cellulose membrane *M*_w_ 3.5 kDa, Thermo, Spain). The obtained polymer conjugate (TAMRA-protamine) was freeze-dried, and NCs were prepared according to the procedure described above.

#### Preparation of PGA–PEG-Enveloped C12-R8 Insulin Nanocomplexes (ENCP)

The nanocomplexes (NCP) were prepared with insulin and C12-R8, making use of hydrophobic and ionic interactions.^13,13,61^ Briefly, C12-R8 was dissolved in water at a concentration of 1 mg/mL. Insulin (insulin glulisine, *M*_w_ 5823 Da, Sanofi Pharma, France) was dissolved at a concentration of 1 mg/mL at 0.01 N NaOH. The NCPs were formed instantly upon mixing the solutions under magnetic stirring at an insulin/R8 molar ratio of 1:8. Blank controls were prepared by adding 0.01 N NaOH solution to a C12-R8 solution to confirm the absence of nanocomplex formation. In a second step, the cationic NCPs were coated by anionic diblock PGA–PEGs, at insulin/PGA–PEG mass ratio 1:0.7, leading to the formation of PGA–PEG-enveloped nanocomplexes (ENCP). The envelopment was done by the film hydration method, where PGA–PEG polymers were dissolved in water, and then, the water was evaporated in a round flask under reduced pressure at 37 °C, resulting in a thin film, followed by the addition of NCP to the same flask and 10 min of rotation at room temperature and atmospheric pressure. Finally, the pH of the ENCP suspension was adjusted to 7.0 with HCl. Fluorescent ENCP were produced using the same method substituting FITC-C12-R8 for C12-R8.

#### Physicochemical Characterization of NP

Particle size distribution and polydispersity index (PDI) were determined by dynamic light scattering (DLS), and ζ-potential was calculated from the electrophoretic mobility values determined by laser Doppler anemometry (LDA). These parameters were obtained with a Malvern Zeta-sizer device (NanoZS, ZEN 3600, Malvern Instruments, Worcestershire, UK) equipped with a red laser light beam (λ = 632.8 nm). To measure the particle size and PDI of PARG NC, 50 μL formulations were diluted with 950 μL of ultrapure water, while those of ENCP were determined directly without any dilution. PrNC and PSA-PrNC were diluted 50× before measurements. For the ζ-potential measurements, the PARG NC, PrNC, and PSA-PrNC samples were diluted with 1 mM KCl solution in the same proportions as before, while the ENCP were mixed with 1 mM KCl, 1:1. The analysis was performed at 25 °C with at least three different batches; each batch was analyzed in triplicate.

#### Association Efficiency (AE) of Insulin

The association efficiency (AE) of insulin in PARG NC was determined upon separation of the NCs from the suspending aqueous medium via ultracentrifuge (Beckman Coulter, Optima L-90K, Brea, CA) at 82 656*g* for 1 h at 15 °C. The AE of insulin for PrNC and PSA-PrNC was determined after isolation of the NC by ultracentrifugation at 80 000*g* for 1 h at 15 °C (Avanti J-E, Ultracentrifuge, Beckman Coulter, Brea, CA). The AE of insulin in ENCP was determined following separation of the complexes from suspension media by centrifugation (Hettich, Universal 32R, Germany) at 15000*g* for 15 min at 15 °C.

The amount of free insulin in the supernatants was determined by reverse phase HPLC (Agilent model 1100 series LC and a diode-array detector set at 214 nm) using a C18 column (Supersphere RP-18 end-capped). A buffer of phosphoric acid and sodium perchlorate was mixed with acetonitrile (93:7 as phase A and 43:57 as phase B, both at pH 2.3). The column was set at 35 °C, and the injection volume was 10 μL. Calibration curves ranging from 5 up to 1050 μg/mL (*r*^2^ = 0.999) were obtained. The limit of quantification (LOQ) and limit of detection (LOD) were 200 and 80 μg/mL, respectively. Each sample was assayed in triplicate.

The AE of insulin in the formulation was calculated taking into account the total insulin amount involved in the formulation and the free insulin found in the supernatant. The final loading was calculated dividing the amount of insulin associated (AE × total insulin in the formulation) by the theoretical amount of all the components involved in the formulation. The final insulin loading (wt %) was calculated by dividing the amount of insulin associated (AE × total insulin in the formulation) by the total weight of the freeze-dried formulations.

#### Electron Microscopy

Scanning transmission electron microscopy (STEM) was used to analyze the shape and surface properties of the NP. For STEM analysis, 10 μL of the diluted samples was deposited on a copper grid for 5 min; excess was removed, and samples were allowed to dry. Micrographs were recorded in the microscope (Ultra Plus and Gemini-500, Zeiss, Germany) with an acceleration voltage of 20.00 kV at different magnifications (50 000× and 100 000×) using a STEM detector.

The morphology of ENCP was analyzed by field emission scanning electron microscopy (FESEM; Gemini-SEM, Zeiss, Germany). 10 μL of the 1:100 diluted samples was deposited on a carbon tape and kept in the desiccator overnight. Samples were coated with iridium in an argon atmosphere. Micrographs were recorded with an acceleration voltage of 3.00 kV at different magnifications (50 000×, 100 000×, or 200 000×) using both SE2 and immersion lens (In-Lens) detectors.

#### Caco-2 Cell Culture

Caco-2 cells were cultured in Dulbecco’s modified Eagle medium (DMEM) supplemented with l-glutamine (1%), heat inactivated fetal bovine serum (10%), penicillin/streptomycin (1%), and nonessential amino acids (NEAA) (1%). Cells were maintained at 37 °C in a humidified incubator supplied with 10% CO_2_. Caco-2 cell monolayers on 12-well cell culture inserts (Transwell, Corning) were used. Caco-2 cell monolayers were formed by seeding 5 × 10^5^ per well which were allowed to grow for 19–21 days ensuring the formation of a tight and fully differentiated columnar monolayer.

#### *In Vitro* Cellular Interaction Study

The interactions of the NP with Caco-2 cells were investigated by confocal laser scanning microscopy. For microscopy, fluorescently labeled nanoparticles were incubated with the cell monolayer for 2 h at 37 °C at a final concentration (mg/mL) of PARG NC (0.37); PrNC (1); PSA-PrNC (1); and ENCP (0.2). After the incubation period, the cell monolayers were washed and fixed using 4% PFA. For DiD-PARG NC, TAMRA-PrNC, and PSA-PrNC, and FITC-labeled ENCPs, actin was stained with 200 μL of Alexa 488-phalloidine and rhodamine-phalloidin, respectively, which were prepared at a ratio of 1:50 in 0.2% (v/v) Triton X-100 in HBSS buffer. The cells were incubated with phalloidin for 10 min in the dark to reveal cell borders, and DAPI (0.5 mg/mL) was used to stain the nucleus, as described. Subsequently, inserts were washed in HBSS, cut, and mounted on glass slides using Mowiol. Images were captured using a Zeiss confocal microscope (LSM 150). Data were analyzed by the Axio Vision software (vs 4.8).

### *Ex Vivo* Characterization of NPs in Ussing Chambers with Human Jejunal Tissue

#### Human Jejunal Tissues

Samples of jejunum were collected from morbidly obese subjects undergoing Roux-en-Y gastric bypass surgery at the Uppsala University Hospital, Uppsala, and the Vrinnevi hospital, Norrköping, Sweden. Subjects followed a low-calorie diet for 3 weeks prior to surgery and did not suffer from inflammatory or infectious bowel diseases at the time of surgery. Subjects diagnosed with type I or II diabetes were excluded from the study. Samples were taken 60 cm from the ligament of Treitz. The study was reviewed and approved by the regional ethical review boards of Uppsala (Approval DNR 2014/531, approval date 2015-03-18) and Linköping (Approval DNR 2013/472-31, Addendum 2015/44-32, approval date 2015-11-20), Sweden. All donors gave written informed consent. Donor characteristics are listed in Table S2.

Tissue samples were put in ice-cold, oxygenated Krebs–Henseleit buffer (KHL, supplemented as described^62^) for transport and were rapidly conveyed to the lab.

#### Ussing Chamber Experiments

Jejunal mucosa was isolated along the mucosa muscularis and mounted in horizontal (homemade) or vertical (Harvard Apparatus, Holliston, MA) Ussing chambers. Tissues were pre-equilibrated for 40 min at 37 °C with two buffer changes. In the chambers carbonate-buffered KHL (pH 7.4) supplemented with 12 mM mannitol was used on the mucosal side while the buffer in the serosal chamber was supplemented with 12 mM glucose.^62^ Chambers were oxygenated and pH stabilized by continuous bubbling with carbogen gas (95% O_2_, 5% CO_2_). Electrophysiology was monitored during the entire experiment.

After pre-equilibration, test compounds or NPs (Table 2) were added to the mucosal chamber together with 4 kDa fluorescein isothiocyanate-labeled dextran (FD4, predialyzed, ThermoFisher) used as a paracellular permeability marker. In experiments with FITC-labeled NPs, 10 μM atenolol was used as a paracellular permeability marker. Samples were taken at intervals from both chambers during the run of the experiment that typically lasted for 120 min. After 120 min 7 μM of the adenylate cyclase activator forskolin was added to the chambers, and the electrophysiological response was monitored to evaluate the continued viability of the tissues.

Ussing chamber electrophysiological quality controls and statistics are shown in Figure S2.

NP were added to three parallel chambers with tissues from three or more donors (on at least three separate experimental days). Size and ζ-potential of all NP batches were determined using DLS after receipt in the Ussing laboratory to ensure that the values corresponded to values determined at NP synthesis. Concentrations of the tested NP were based on toxicity data from Caco-2 experiments and are listed in Table 2.^11−13^ After initial experiments showed a lack of toxicity for PARG NC, PrNC, PSA-PrNC, and ENCP, a higher concentration of these NP was also tested. PARG NC were only used in a blank, unloaded form devoid of insulin as insulin-loaded PARG NC consistently showed rapid aggregation and sedimentation in the Ussing chambers. Doses of PrNC and PSA-PrNC were limited by the maximum achievable stable nanoparticle concentration in the stock solution. As a control, the permeability and toxicity of NP constituents (free TAMRA-protamine and FITC-C12-R8 polymer) without incorporation into NP were tested in Ussing chamber experiments. Doses used corresponded to the amount of material added in nanoparticle experiments. As a further control fluorescently labeled 200 nm polystyrene NP (Fluospheres, ThermoFisher) with aminated cationic surfaces (PS-NP+) or carboxylated anionic surfaces (PS-NP−) were used.

Samples of fluorescently labeled NP or FD4 were analyzed in a plate reader (Saffire^2^, Tecan) and the apparent permeability constant (*P*_app_) was calculated as described.^33,63^ The LOQs of the fluorescent measurements were typically at the level of 1/10 000 of the NP concentration used in the Ussing chambers. Samples containing pharmacological probes or insulin-loaded nanoparticles were analyzed by UPLC-MSMS as described below.

To ensure that measured permeability of fluorescently labeled NPs was not due to released free fluorophores, monitoring of NP integrity after permeation of the mucosa was performed using Spectra-Por Float-A-Lyzer dialysis devices with a molecular weight cutoff of 3.5–5 kDa (Spectrumlabs). Samples (500 μL) were taken from both apical and basolateral chambers at the end of experiments and dialyzed for 48 h against 2 L of PBS at 4 °C with stirring. PBS was refreshed every 24 h. Diluted nanoparticle stocks not exposed to tissue were dialyzed in parallel as controls. Fluorescence remaining in the dialysis device after 48 h was quantified and compared to values before dialysis.

#### Endocytotic Pathways

The involvement of active endocytotic pathways in jejunal NP permeability was investigated using chemical inhibitors of endocytosis. Tissues were pretreated with the dynamin inhibitor dynasore (Sigma-Aldrich) at 80 μM for 15 min before addition of NP. As a control for endocytosis, a 3 kDa lysine-fixable fluorescently labeled dextran (FD3, ThermoFisher) was used. Inhibition of transcytotic permeability was tested using horseradish peroxidase (HRP, Sigma-Aldrich). Permeabilities of NP and HRP were measured, and after the experiment, internalization of nanoparticles and FD3 was investigated by microscopy. HRP permeability was analyzed by ELISA as previously described.^36,62^

#### UPLC-MSMS Analysis

Samples containing pharmacological probes were analyzed on an ACQUITY UPLC (Waters) instrument linked to a Xevo triple-quadrupole tandem mass spectrometer (Waters). LC and MS methods were optimized for each compound. Results were quantified using TargetLynx software (Waters).

#### Insulin Analysis

Permeated insulin was analyzed by HPLC-MSMS. LC (Shimadzu HPLC system LC 20AD, Japan) with a 150 × 2.1 mm–5 μm–300 Å HPLC C8 column (Interchim, France) was used for the elution of insulin with a gradient method. Mobile phases A and B were 0.1% formic acid solution and acetonitrile containing 0.1% formic acid, respectively. The flow rate was 0.6 mL/min. 100 μL of the tested sample was treated with 200 μL of chloroform/methanol/water at 1/1/0.3 and 100 μL of 0.1 M NaOH, and then, 40 μL of analyte was injected onto the column placed in an oven at 60 °C. The total run time was 13 min. Tandem mass spectrometry in positive electrospray mode was used for detection. System control and data processing were carried out using MassLynx software version 4.1. Spray voltage was 3.0 kV, and sheath and auxiliary gas pressures were 50 and 15 (arbitrary units), respectively. The in-source CID energy was fixed at 12 V, and capillary temperature was 350 °C. Tube lens and collision energy values were optimized for insulin. Multiple reaction monitoring (MRM) was used for the detection of the ion transitions. MRM transitions for analytes were as follows: *m*/*z* insulin 709.805 N 731.76, *m*/*z* insulin 1284.73 N 1104.60. Analytes were quantified by means of calibration curves using insulin as internal standard. The standard curves showed linearity over a range of 0.025–10 μg mL^–1^ for insulin. This represents an LOQ of 1/1000 of the lowest insulin dose administered in the Ussing experiments.

#### Modeling of Jejunal Fraction Absorbed

Human jejunal fraction absorbed was predicted from *ex vivoP*_app_ values using a simple computational model incorporating luminal volume, epithelial surface area, and jejunal transit time.^32^

#### Lactate Dehydrogenase (LDH) Release

Cytotoxicity of NP was assessed by monitoring the induced release of LDH from human jejunal mucosa in the Ussing chambers. Samples were taken at intervals from the mucosal side (to which NP were added) of three parallel NP-containing chambers and three untreated negative control chambers. As a positive control the control chambers were at the end of the experiment treated with 5% Triton X-100 (Sigma-Aldrich) for 10 min. Experiments were repeated with at least two donors. The amount of released LDH was measured using the cytotoxicity detection kit plus LDH (Roche) according to the manufacturer’s instructions.

#### ATP Levels in Human Jejunal Tissue

Cytotoxicity of NP was also assessed by monitoring tissue ATP levels. After a 2 h exposure of tissues to NP, tissues were removed from the chambers and snap frozen. Samples were stored at −150 °C until analyzed. Tissues from three parallel NP treated samples and three untreated control samples were collected from experiments with two or more tissue donors per NP. Tissue pieces of 5 mg were homogenized and sonicated rapidly on ice in 50 μL of DMEM (GIBCO). ATP concentrations in the lysates were determined using the CellTiter-Glo kit (Promega) according to the manufacturer’s instructions. ATP concentrations were normalized to protein content of the tissue samples determined by the tryptophan fluorescence method.

#### Multiplex ELISA of Inflammatory Biomarkers

To investigate potential inflammatory responses of the human intestinal epithelium to NP treatment, samples were collected from basolateral chambers at regular time intervals up to 120 min. Samples were collected from triplicate chambers from experiments with the highest concentrations of NP, and triplicate untreated chambers were included on each occasion as negative controls. As a positive control LPS (50 ng/mL) was added to the apical side of triplicate chambers. A baseline sample at *t* = 0 was collected from every chamber. Samples were snap frozen in liquid nitrogen and stored at −150 °C until analysis. Samples were then analyzed for concentrations of cytokines released from the tissue.

A protease inhibitor cocktail was prepared, consisting of 5.5 μL of 10 mM KR-62436^64^ (DPP4 inhibitor) and a SIGMAFAST protease inhibitor tablet (both produced by Sigma-Aldrich Corp., St. Louis, MO, cat. K4264) dissolved in 2100 μL of distilled water (∼50× stock) as done previously in preparing human plasma/serum.^65−67^ Inhibitor tablets contained the following protease inhibitors (mM final concentrations): AEBSF 2; phosphoramidon 1; bestatin 130; E-64 14; leupeptin 1; aprotinin 0.2; and pepstatin A 10. Prior to thawing Ussing chamber samples (approximately 120 μL/tube), 2 μL of 50× protease inhibitor cocktail was added. Once thawed, samples were vortexed and analyzed for inflammatory markers by validated multiplexed ELISA using electrochemiluminescence detection. Samples were distributed onto 3 different 96-well multispot 10-plex plates (human cytokine 30-plex, cat. K15054G-2, Meso Scale Diagnostics, Rockville, MD). Two plates had IL-8 with different standard ranges; hence, there were 29 different analytes total. Samples were diluted 2-fold (cytokine and proinflammatory panel plates) or 4-fold (chemokine panel plates) according to the manufacturer’s recommendations. The Meso Scale Diagnostics QuickPlex SQ120 imager was used to read plates. The 29 inflammatory markers investigated were as follows [analyte, LLOQ in ng/L (CV%)]: (1) eotaxin 11.5 (2.4); (2) eotaxin-3 10.2 (4.6); (3) IP-10 1.37 (6.7); (4) MCP-1 0.99 (1.3); (5) MCP-4 4.91 (1.1); (6) MDC 20.5 (1.4); (7) MIP-1α 7.91 (3.2); (8) MIP-1β 2.13 (1.6); (9) TARC 0.76 (1.5); (10) IFN-γ 3.13 (4.3); (11) IL-10 0.191 (5.1); (12) IL-12p70 0.836 (5.3); (13) IL-13 1.01 (4.3); (14) IL-1β 1.08 (5.3); (15) IL-2 0.89 (5.3); (16) IL-4 0.126 (5.3); (17) IL-6 0.457 (4.8); (18) IL-8 0.333 (3.1); (19) TNF-α 0.664 (4.5); (20) GM-CSF 1.56 (5.2); (21) IL-12/IL-23p40 5.05 (2.4); (22) IL-15 1.12 (4.9); (23) IL-16 4.62 (1.8); (24) IL-17A 9.32 (5.3); (25) IL-1α 2.75 (4.1); (26) IL-5 1.34 (5.1); (27) IL-7 1.03 (3.3); (28) TNF-β 0.955 (5.3); and (29) VEGF 1.84 (2.1). Concentrations are given after correction for sample dilution.

#### Fluorescence Microscopy, Laser Scanning Microscopy (LSM), and Super-Resolution Structured Illumination Microscopy (SIM)

Human jejunal samples from Ussing chambers were taken after 90 min of permeability experiment and fixed, still mounted in the chambers, with phosphate-buffered 4% paraformaldehyde at 4 °C overnight and rinsed. Samples were transferred to 30% sucrose in PBS (w/v) for 48 h, mounted in OCT Cryomount (HistoLab) and sectioned. Cryosections were cut to a thickness of 8 μm on a Leica CM1950 cryostat (Leica). Cryosections were mounted on slides, stained, and used for histology, epifluorescence microscopy, or LSM. For SIM (and some light microscopy imaging) imaging tissue was paraffin embedded, cut in 4 μm sections on a microtome, and used for super-resolution imaging by SIM.

To localize nanoparticle interactions with jejunal tissue, 8 μm cryosections were counterstained with combinations of DAPI (0.5 mg/mL, nuclear stain, ThermoFischer), fluorescently labeled phalloidin (0.13 μM, actin stain, ThermoFisher), and fluorescently labeled wheat germ agglutinin (4 μg/mL, membrane stain, ThermoFisher). Fluorophore labels were selected to not interfere with the fluorescent signal from the labeled NP. Coverslips (High Precision No. 1.5H, Paul Marienfeld GmbH, Lauda-Königshofen, Germany) were mounted with Prolong Diamond Antifade mounting medium (ThermoFisher). Images were acquired on a Zeiss Elyra S.1 SIM instrument in LSM mode and Zeiss BF/LF Axioimager microscopes at the Uppsala university core facility Biovis. Images were deconvoluted using Huygens software and processed using ZEN (Zeiss) and IMAGE J software. Quantitation of cells and NPs was performed using ImageJ.

To evaluate NP interaction with tissues, five sections from each of two tissue specimens from two parallel Ussing chambers were stained, imaged, and analyzed. The process was repeated in at least two experiments with tissues from different donors.

#### Super-Resolution Microscopy of NP in Human Jejunal Tissues

Tissue samples were fixated with phosphate-buffered 4% formaldehyde in the Ussing chambers overnight followed by sucrose treatment, dehydration, embedding in paraffin, and sectioning. Sections of 4 μm were mounted as above and used for structured illumination super-resolution microscopy (SIM) of fluorescently labeled nanoparticles using a Zeiss Elyra S.1 SIM microscope. Images were processed as described above.

#### Quantitation of NP in Human Jejunal Tissues

Images of NP in jejunal mucosa were acquired as dense *z*-stacks using SIM. Nanoparticles visible on images were counted using ImageJ and designated as residing in the brush-border membrane, in the enterocyte, or in the subepithelial space. Using length and *z*-thickness of the image, the perpendicular surface area of the imaged jejunal epithelium could be calculated. Using common scaling factors for jejunal surface amplification by villi (8.6 times) and mucosal folding (3 times),^48^ the number of absorbed nanoparticles per Ussing chamber could be estimated and used for predictions of nanoparticles absorbed during a jejunal transit.

#### Microscopy of Rat *in Vivo* Intestinal Samples

Rat jejunal loops were exposed to NP in *in situ* experiments described below. After the experiment jejunal loops were fixed in 10% (w/v) formalin and subsequently embedded in paraffin. 5 μm tissue sections were cut on a microtome (Leitz 1512; GMI), mounted on adhesive coated slides, stained with hematoxylin/eosin (H&E) and Alcian blue, and examined under light microscopy at 4× magnification (Nikon Labphoto; Nikon, Japan). The interaction of fluorescently labeled NPs (DID-labeled PARG NC, TAMRA-labeled PrNC, and FITC-labeled ENCP) with rat jejunal tissue was carried out *in vivo* using the intrajejunal instillation model over 2 h with no blood sampling. Posteuthanasia, loops were preserved in 10% formalin and embedded in paraffin. 5 μm sections were cut and mounted on electrostatically charged slides (Superfast Ultra, Thermo-Fisher). Images were acquired using a Zeiss Axioplan microscope (20× objective).

### *In Situ* Experiments—Intestinal Instillation in Rat Jejunum

#### Animal Handling and Surgical Procedures

Intrajejunal instillations were performed, as previously described^23,24,68,69^ with minor modifications, under license AE18982/P036 from the Irish Health Products Regulatory Authority (HPRA) and with the approval of the University College Dublin (UCD) Animal Research Ethics Committee (protocol AREC 13-40-Brayden). Nondiabetic male Wistar rats (Charles River Laboratory, UK, and UCD Biomedical Facility) weighing approximately 340 g were randomly selected for experiments. Animals were housed under controlled conditions of temperature and humidity with a 12 h/12 h light/dark cycle. Rats received filtered water and standard laboratory chow ad lib and were fasted 16–20 h prior to the procedure.

All procedures were carried out under anesthesia (Iso-Vet, 1000 mg/g isoflurane liquid for inhalation, Piramal Healthcare, UK). Briefly, following a midline laparotomy, a 5–7 cm loop was created in the jejunum with a size 4-braided silk suture. NP solutions were injected into the lumen using a 1 mL syringe with a 30G needle. NP doses were normalized to contain 50 IU insulin.

Integrity of jejunal tissues after administration of the same volume of PBS and levels of autofluorescence in rat jejunal tissue section are shown in Figure S6.

#### Serum Glucose and Insulin Measurements

Glucose levels were determined with blood obtained from the tail vein and measured using a glucometer (Accu-chek Aviva, Roche). Retro-orbital blood samples were taken and stored at 2–8 °C prior to centrifugation (6500*g*, 5 min) and serum collection. Serum was stored at −20 °C until analysis. Serum Insuman levels were quantified using a Human Insulin ELISA (Mercodia, Sweden). PBS and 50 IU/kg insulin solutions were used as controls. Plasma levels of insulin glulisine were measured by an exploratory LC-MS/MS method at Sanofi (Frankfurt, Germany). For calculation of mean concentrations, values below the lower limit of quantification (LLOQ = 0.1 ng/mL in plasma) were set to zero.

#### Statistics

Statistical analysis of *in vitro*, and *ex vivo* experiments was carried out using Prism-5 software (GraphPad, San Diego, CA) using Student’s *t* test or two-way ANOVA.

Statistical analysis of *in situ* experiments was carried out using Prism-5 software (GraphPad, San Diego, CA) using two-way ANOVA with Bonferroni’s post-test.

Results are presented as mean ± standard deviation (SD); significant differences were defined as *p* < 0.05.

## Supporting Information Available

NP toxicity in the human jejunum; NP toxicity data from isolated human jejunal mucosae in Ussing chambers; data on cytokines released from jejunal tissues in Ussing chambers; characteristics of jejunal tissue donors; assessment of NP stability during permeability of the jejunal mucosa; electrophysiology and FD4 permeability in Ussing chambers; NP induced LDH release from human jejunal tissue; NP interactions with Caco-2 cells grown on Trans-well filters, images indicating that free FITC-C12-R8 and Tamra-Protamine do not show mucus binding; quantitation of NP mucus and tissue binding, image showing permeation of PrNC into jejunal mucosa below defect in epithelium; effects of dynasore on PrNC permeability across human jejunal mucosa; area above the blood glucose concentration–time curve (AAC) for the tested NP after rat jejunal instillation; and histology from rat *ex vivo* studies (PDF)
