# Supplementary material for: Barriers to the Intestinal Absorption of Four Insulin-Loaded Arginine-Rich Nanoparticles in Human and Rat
Source: ACS Nano. 2022 Aug 23;16(9):14210–29. doi: 10.1021/acsnano.2c04330 (PMC9527806; doi:10.1021/acsnano.2c04330)
Supplement: Supplementary file 1 — nn2c04330_si_001.pdf [file nn2c04330_si_001.pdf]

Supporting information

Barriers to the intestinal absorption of four insulin-loaded arginine-rich nanoparticles in human and rat.

Patrik Lundquist[1], Georgiy Khodus[1], Zhigao Niu[2], Lungile Nomcebo Thwala[2,3], Fiona McCartney[4,], Ivailo Simoff[1], Ellen Andersson[5, 6], Ana Beloqui[3], Aloise Mabondzo[7], Sandra Robla[2], Dominic-Luc Webb[8], Per M. Hellström[8], Åsa V Keita[6], Eduardo Sima[9], Noemi Csaba[2], Magnus Sundbom[9], Veronique Preat[3], David J. Brayden[4,] , Maria Jose Alonso[2], and Per Artursson[1]

1. Department of Pharmacy, Uppsala University, SE-751 43, Uppsala, Sweden.
2. CIMUS, Department of Pharmacy and Pharmaceutical Technology, Universidade de Santiago de Compostela, Santiago de Compostela, ES 15782, Spain.
3. Université catholique de Louvain, UCLouvain, Louvain Drug Research Institute, Advanced Drug Delivery and Biomaterials, BE 1200, Brussels, Belgium.
4. UCD School of Veterinary Medicine, University College Dublin, Belfield, D04 V1W8, Ireland
5. Department of Surgery in Norrköping, Linköping University, Norrköping, SE-581 83, Sweden.
6. Department of Biomedical and Clinical Sciences, Linköping University, SE-581 83, Linköping, Sweden.
7. CEA, Institute of biology and Technology of Saclay, Department of Pharmacology and Immunoanalysis, Gif sur Yvette, FR 91191, France
8. Department of Medical Sciences, Uppsala University, SE-751 85, Uppsala, Sweden.
9. Department of Surgical Sciences - Upper Abdominal Surgery, Uppsala University, SE-751 85, Uppsala, Sweden.

Table of contents

Page

3 - NP toxicity in the human jejunum
4 - Supplementary Table S1. NP toxicity data from isolated human jejunal mucosae in Ussing chambers.
5 - Supplementary Table S2. Cytokines released from jejunal tissues in Ussing chambers.
6 - Supplementary Table S3. Characteristics of jejunal tissue donors.
7 - Fig.S1 Assessment of NP stability during permeability of the jejunal mucosa.
8 - Fig.S2 Electrophysiology and FD4 permeability.
9 - Fig. S3. NP induced LDH release from human jejunal tissue.
10 - Fig. S4. NP interactions with Caco-2 cells grown on Trans-well filters.
11 - Fig. S5. Free FITC-C12-R8 and Tamra-Protamine do not show mucus binding.
12 - Fig. S6. Quantitation of NP mucus and tissue binding.
13 - Fig. S7. Permeation of PrNC into jejunal mucosa below defect in epithelium.
14 - Fig. S8. Effects of dynasore on PrNC permeability across human jejunal mucosa.
15 - Fig. S9. Area above the blood glucose concentration – time curve (AAC) for the tested NP after rat jejunal instillation.
16 - Fig. S10. Histology from rat *ex vivo* studies.

NP toxicity in human jejunum. All NP displayed low toxicity to the jejunal tissue at a concentration of 0.2 – 5.8 mg/ml (Supplementary table 1). No effect on tissue electrophysiology, viability or ATP-levels were observed during the Ussing chamber experiments. This is not surprising, given that most NP did not reach the epithelial cell layer in significant amounts. However, ENCP exposure was associated with some release of LDH (Supplementary Fig. S3). This was probably a result of a stronger interaction between ENCP and the epithelial cell surface.

All tested NP showed markedly higher toxicity in Caco-2 cells than in isolated human jejunal tissue in the present study. Thus, the jejunal tissue was unaffected by NP concentrations that reduced cell viability in Caco-2 cell culture. This discrepancy is likely due to the protective effect of the mucus-layer hindering nanoparticle – tissue interactions. The jejunal tissue was also less sensitive to mucus penetrating nanoparticles. ENCP at a concentration of 0.2 mg/ml led to an 80% decrease in viability in Caco-2 cell culture while jejunal tissue remained unaffected at a five times higher concentration (1 mg/ml).

The effects of the NP on cytokine release were also investigated. As many as 29 cytokines were quantified in the jejunal effusates collected from the serosal side of Ussing chambers after 60 min exposure or later time points (Supplementary Table S2). None of the tested NP stimulated a statistically significant increase in cytokine release from the tissues. Positive controls using LPS (50 ng/ml) showed an increased release of several pro-inflammatory cytokines such as TGF-β. This experiment indicates that the tested NP elicited little or no acute inflammatory response in human jejunum *ex vivo*.

	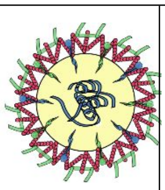	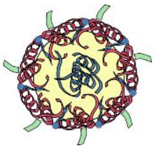	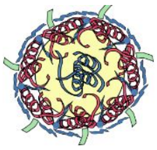	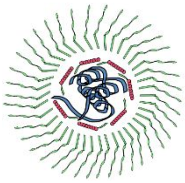	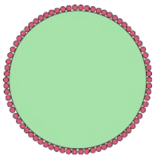	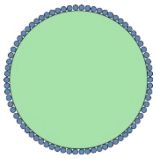
	1	2	3	4	5	6
Nanoparticle	PARG NC	PrNC[e]	PSA-PrNC[e]	ENCP[e]	PS-NP+	PS-NP-
NP Concentration, mg/ml[a]	Low; 1<br>High; 3.2	Low: 1<br>High: 5.8	Low: 1<br>High: 5.8	Low: 0.2<br>High: 1	1	1
Effect on e-physiology[b]	None	None	None	None	None	None
Effect on tissue viability[c]	None	None	None	None	None	None
Induced LDH release[d]	No	No	No	**Yes**	No	No
Effect on tissue ATP levels	No	No	No	No	No	No
Induction of cytokine release	No	No	No	No	No	No

[a]No differences were seen between low and high NP doses. [b]Assessed as effects on average electrophysiological values and [c]forskolin response. [d]See Supplementary Fig. S3. [e]Free TAMRA-protamine and FITC-C12-R8 showed no toxicity in any experiment.

Supplementary Table S1. NP toxicity data from isolated human jejunal mucosae in Ussing chambers.

**Chemokines**									
Eotaxin	Eotaxin-3	IL-8	IP-10	MCP-1	MCP-4	MDC	MIP-1α	MIP-1β	TARC
**Proinflammatory cytokines**									
IFN-γ	IL-10	IL-12p70	IL-13	IL-1β	IL-2	IL-4	IL-6	IL-8	TNF-α
**Cytokines**									
GM-CSF	IL-12/IL-23p40	IL-15	IL-16	IL-17A	IL-1α	IL-5	IL-7	TNF-β	VEGF

Supplementary Table S2. Cytokines released from jejunal tissues in Ussing chambers. Cytokine release was monitored from 0 to 120 min in the basolateral chamber. The listed cytokines could be detected at the 60 min time point and after.

No.	Age	Sex (F/M)	BMI (kg/m^2)
1	44	F	33.1
2	21	F	49.6
3	49	F	43
4	41	F	32.8
5	18	F	41.4
6	34	M	42.3
7	56	F	39.4
8	54	F	41.6
9	48	F	34.5
10	34	F	41.4
11	22	F	34.6
12	24	F	43.1
13	45	M	46.0
14	55	F	37
15	28	F	35.1
16	53	F	38.5
17	53	F	51.9
18	46	F	38.5
19	26	F	33.5
20	45	M	40.7
21	42	M	35.8
22	30	F	34.9
23	60	F	39.8

Supplementary Table S3. Characteristics of jejunal tissue donors.
BMI is listed as measured on the day of surgery.

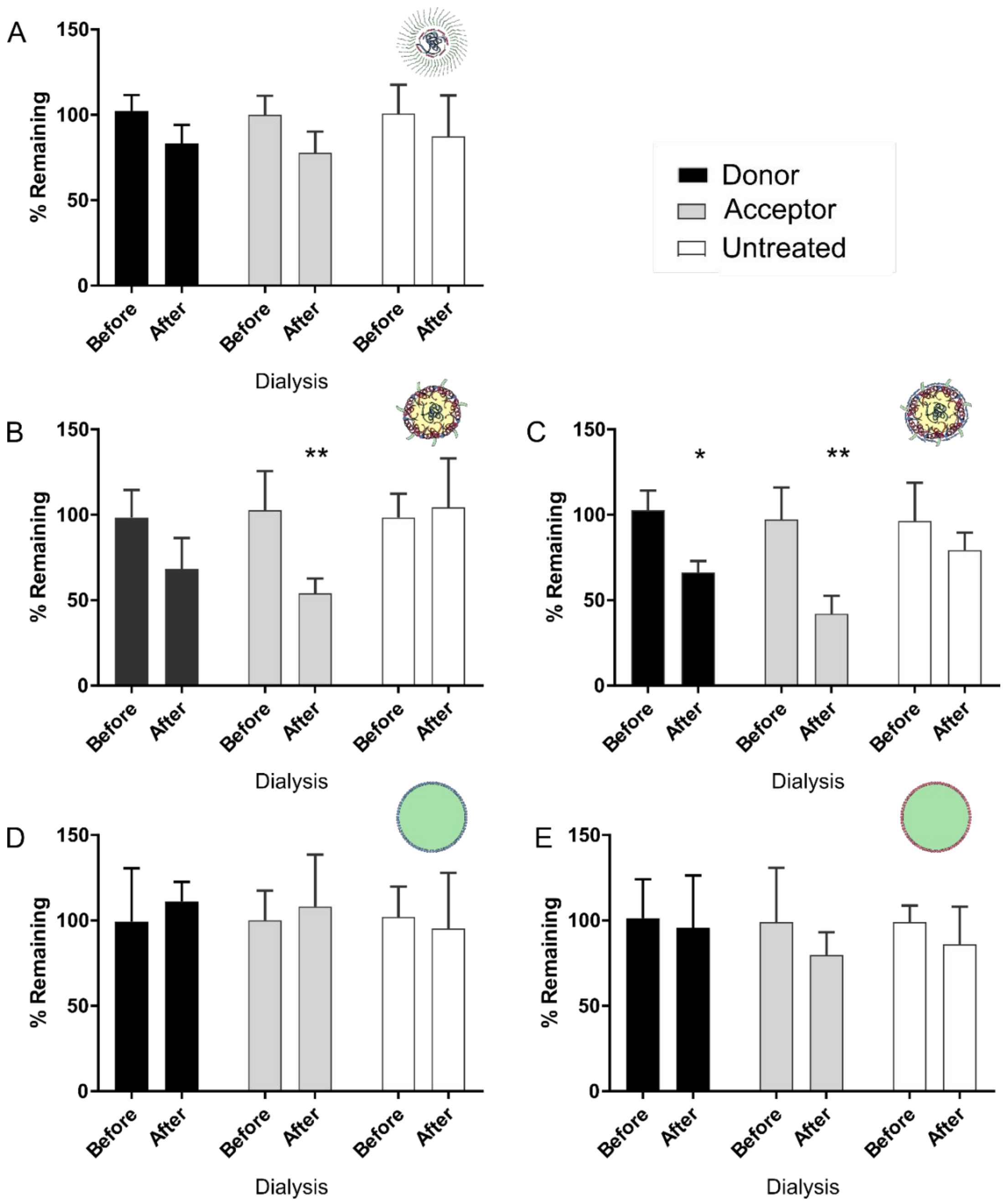

Fig.S1 Assessment of NP stability during permeability of the jejunal mucosa. Dialysis of NP containing samples from donor and acceptor chambers taken at 120 min after addition of NP to Ussing chambers, as well as untreated control samples. **A:** ENCP. **B:** PrNC. **C:** PSA-PrNC. **D:** PS-NP-, **E:** PS-NP+. In the case of ENCP, PS-NP+ and PS-NP- the signal is unchanged before and after dialysis indicating that the fluorophore is connected to material > 3-5 kDa (the MWCO of the dialysis membrane). For PrNC and PSA-PrNC the fluorescent signal in control samples or samples from Ussing donor chambers are generally unaffected after dialysis indicating the presence of intact NP. In samples from Ussing acceptor chambers the fluorescent signal decreases after dialysis, indicating that PrNC and PSA-PrNC partially have been digested during the passage through the jejunal mucosa. Before: Levels of fluorescence in samples before dialysis. After: Levels of fluorescence remaining in dialysis chamber after 48 h. Average ± SD shown, n = 3.

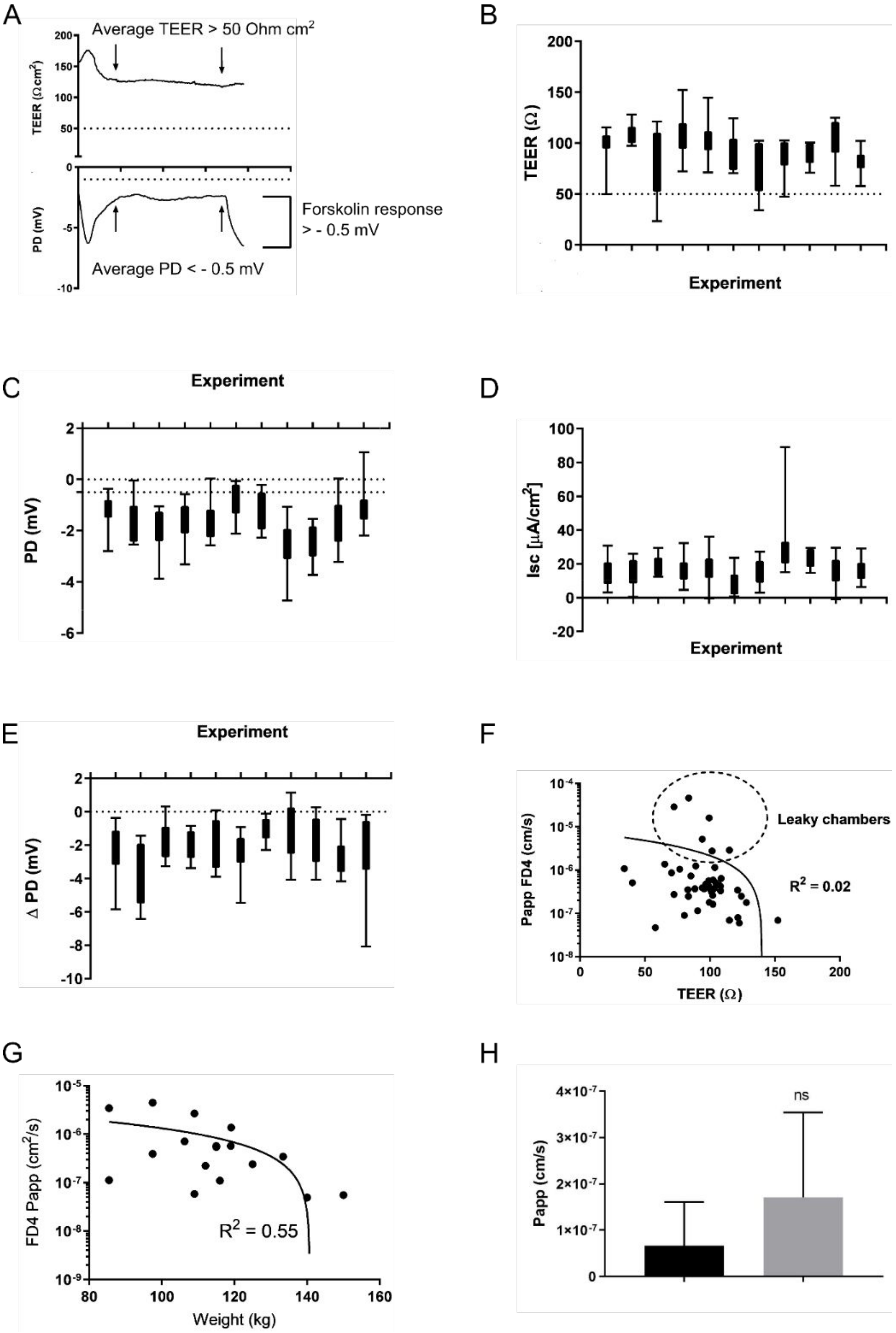

Fig.S2 Electrophysiology and FD4 permeability A: Illustration of electrophysiology parameters showing a good quality sample. Quality control cut-offs are illustrated in the graph. In total 2% of chambers were excluded due to sub-optimal electrophysiological values. 6% of chambers were excluded due to leakage of paracellular permeability markers. **B-D**: Electrophysiology statistics from Ussing experiments with 12 donors, 6-20 chambers per experiment. Quartile groups are shown. (B: Trans-epithelial electrical resistance, TEER; C: Potential difference, PD; D: Short circuit current, Isc.). **E**: Response in PD to forskolin indicated good viability of jejunal tissue in the majority of chambers**.** **F**: Distribution of Papp values for the paracellular permeability marker FD4. Leaky chambers were defined as those displaying a FD4 Papp value of > 3 times the median value. Excluded chambers are indicated in the graph. No clear correlation between FD4 Papp and TEER was seen (linear regression). **G**: Relationship between FD4 permeability and body weight of donors. No increase in permeability was seen with increasing body weight (linear regression). **H**: Increase of FD4 permeability over intestinal epithelia treated with PrNC. Average ± SD shown, n = 6.

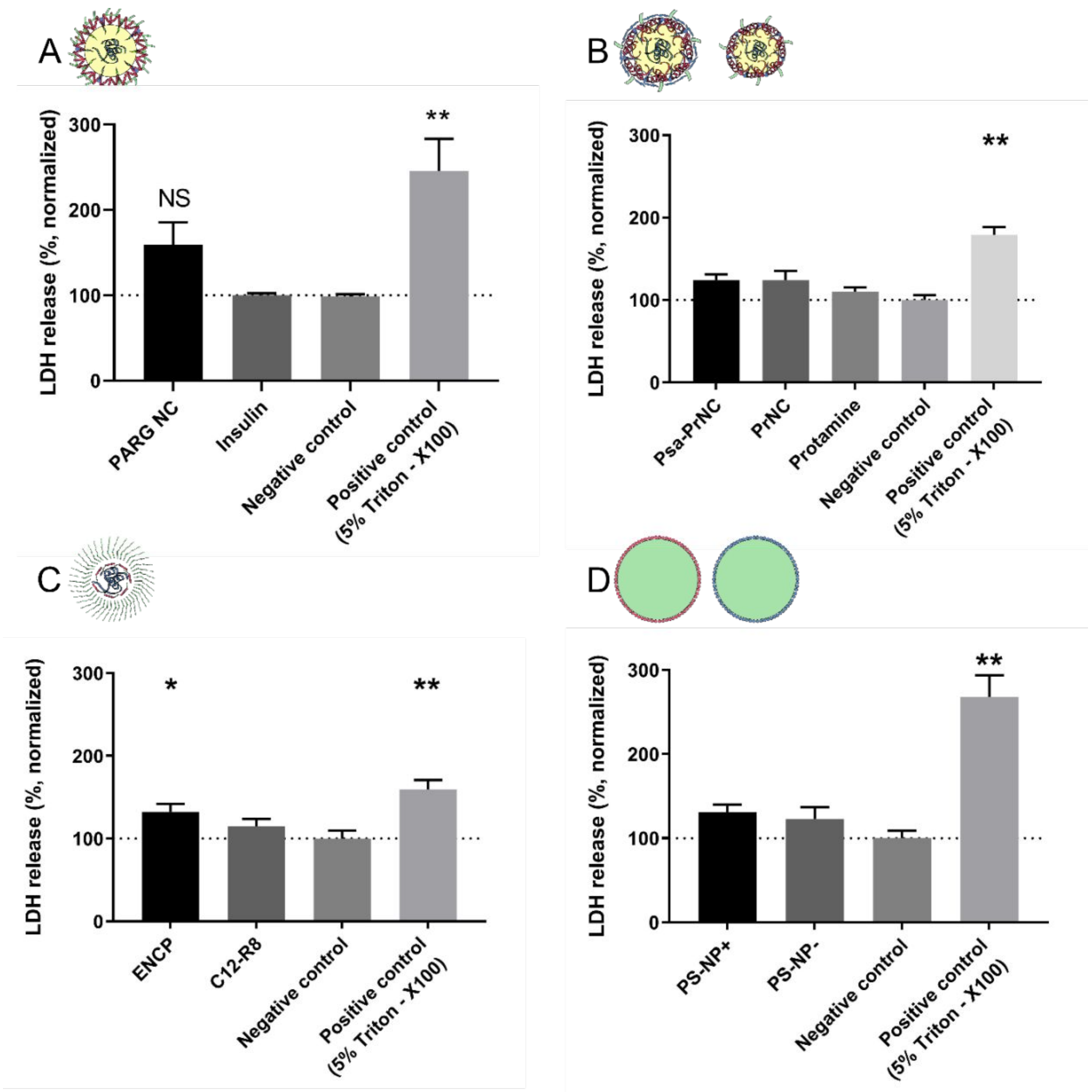

Fig. S3. NP induced LDH release from human jejunal tissue. LDH release from human jejunal tissue was measured after exposure to NP, or NP constituents, in Ussing chambers for 2 h. All NP were tested at the highest concentration given in Supplementary Table S1. **A:** PARG NC. **B:** PrNC and PSA-PrNC. **C:** ENCP. **D:** PS-NP+ and PS-NP-. Only ENCP induced a small LDH leakage from the tissue. Average ± SD shown, n = 3.

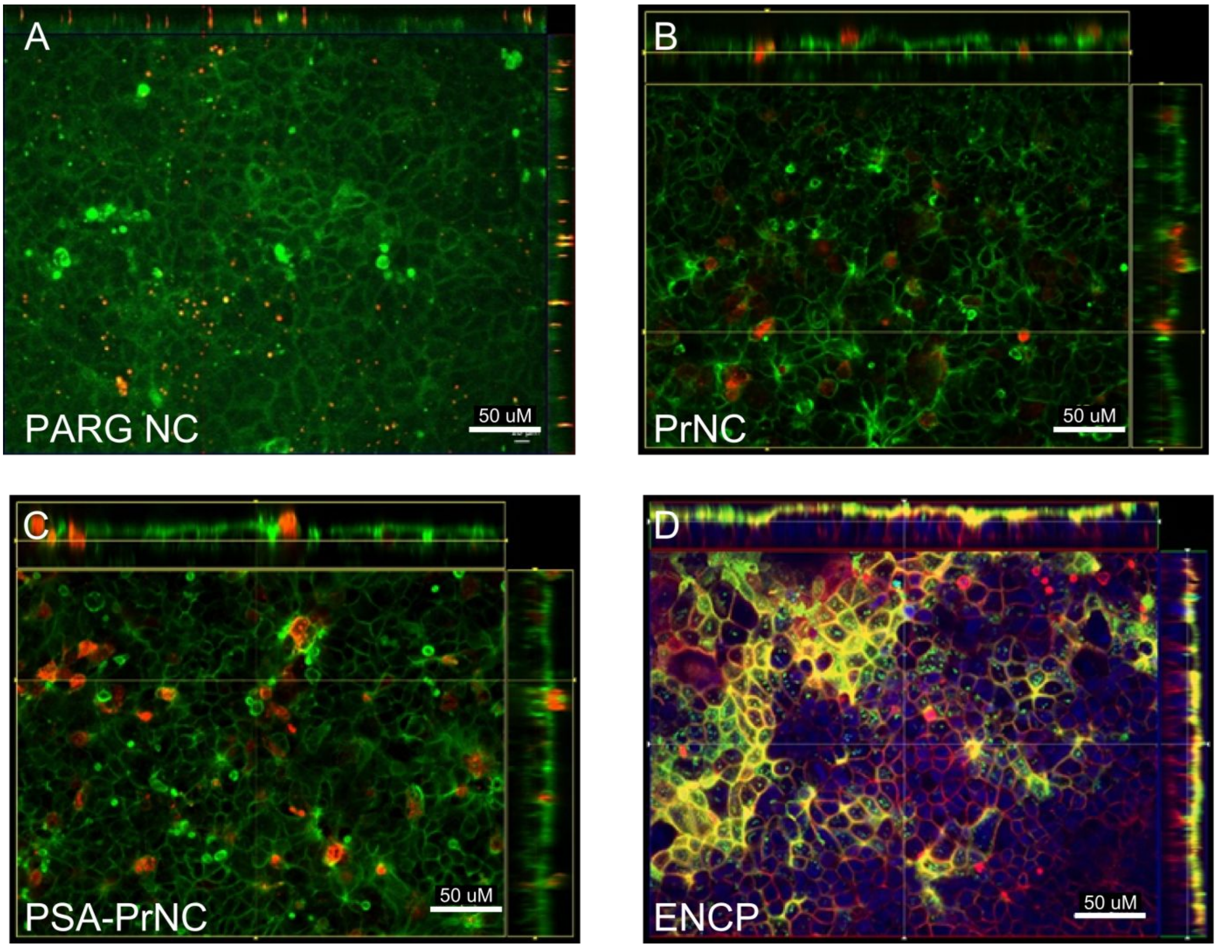

Fig. S4. **NP interactions with Caco-2 cells grown on Trans-well filters. A:** PARG NC; **B:** PrNC; **C:** PSA-PrNC; and **D:** ENCP with Caco-2 cells. PARG, PrNC and PSA-PrNC show little uptake and binding to Caco-2 cells. ENCP were to a large extent taken up by Caco-2 cells and mainly localized within the BBM. Actin staining in green, NP in red.

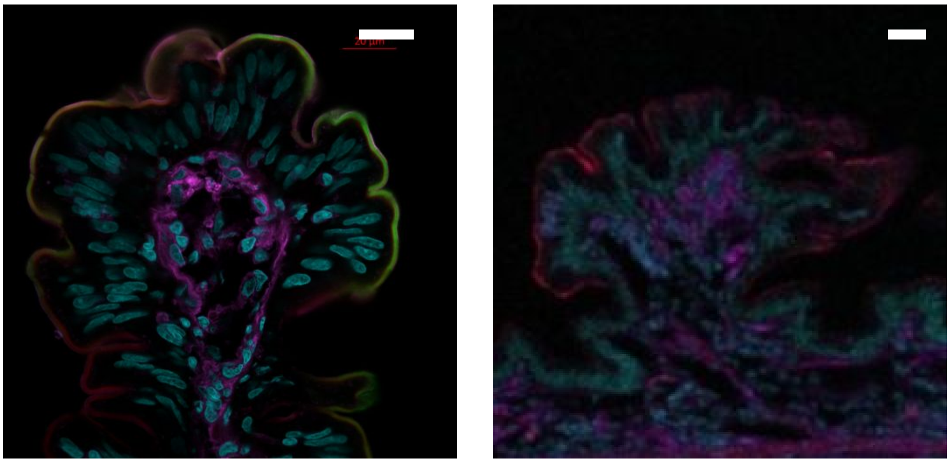

Fig. S5. **Free FITC-C12-R8 and TAMRA-Protamine do not show mucus binding.**
The fluorescently labeled polymers FITC-C12-R8 and TAMRA-Protamine were added to Ussing chambers with human jejunal mucosa in their free form, not being incorporated into NP. **A.** Free FITC-C12-R8 (green) could not be detected in the mucus surrounding the villi but permeated to the epithelium and accumulated in the BBM of jejunal enterocytes (see also Fig. 5E). No FITC-C12-R8 could be detected below the BBM. **B.** Free TAMRA-protamine (green) could not be detected in the mucus layer surrounding the villi. Further, no BBM accumulation was observed. FITC-C12-R8 and TAMRA-protamine are stained green, actin is stained red, cell membranes are stained purple and nuclei are stained cyan. The scale bar (white) depicts 20 µm. Images represent the distribution of fluorophore after 90 min of the Ussing chamber experiment. Images were produced from three sections in tissue specimens derived from at least two parallel Ussing chambers from each of two donors. Stainings were consistent between all images.

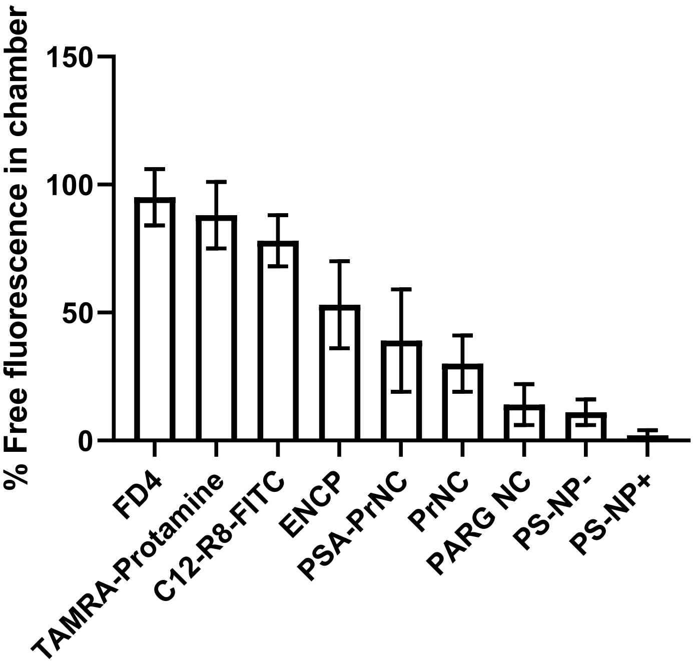

.

Fig. S6. **Quantification of NP mucus and tissue binding.** The levels of fluorescent label remaining in in the fluid in apical Ussing chambers were measured at the start (100 %) and end of the experiment. This gives an estimate of the fraction of NP that has been bound to the tissue specimen in the chamber. No measurable reduction was seen after incubations in empty chambers without tissue. Average ± SD shown, n = 6.

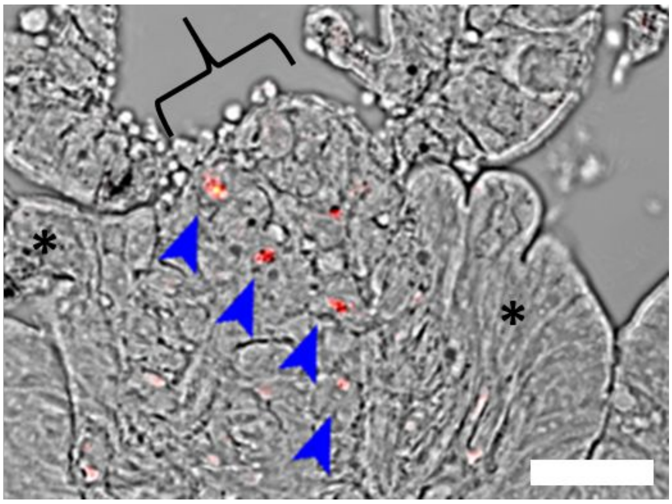

Fig. S7. Permeation of PrNC into jejunal mucosa below defect in epithelium. Human jejunal mucosa, after exposure to PrNC in an Ussing chamber experiment, is shown in a bright field image. PrNC fluorescence is visualized in red-orange and NP are indicated by blue arrows. PrNC are found within the tissue beneath a defect in the epithelium indicated by the black bracket (see also Fig. 3C in the main text). In the indicated area, the epithelial enterocytes are missing but are present to the right and left of the indicated area (asterisk). In intact epithelium the BBM can be seen as a lighter border on the apical edge of enterocytes. The image represents the distribution of NP after 90 min exposure in the Ussing chamber (apical side). The image were produced from three sections in tissue specimens derived from at least two parallel Ussing chambers from each of two donors. Staining was consistent in all images**.** The scale bar (white) depicts 25 µm.

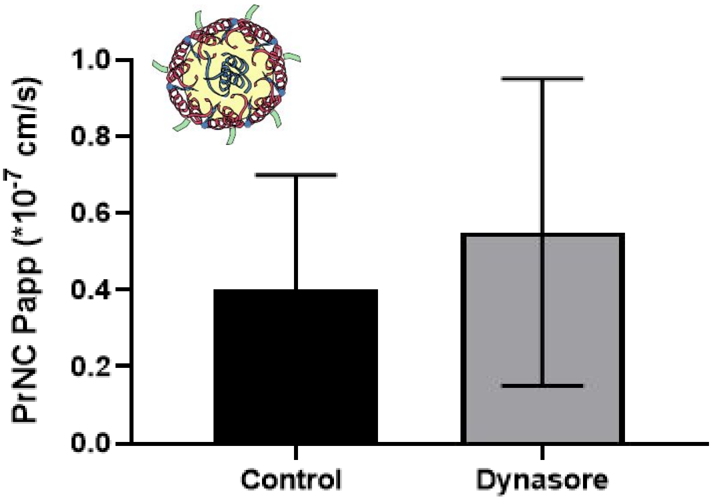

Fig. S8. Effects of dynasore on PrNC permeability across human jejunal mucosa. Permeability of PrNC was measured in human jejunal mucosa mounted in Ussing chambers with and without the addition of the dynamin and endocytosis inhibitor dynasore. Treatment with dynasore had no significant effect on the permeability of PrNC. Average ± SD is shown, n = 6.

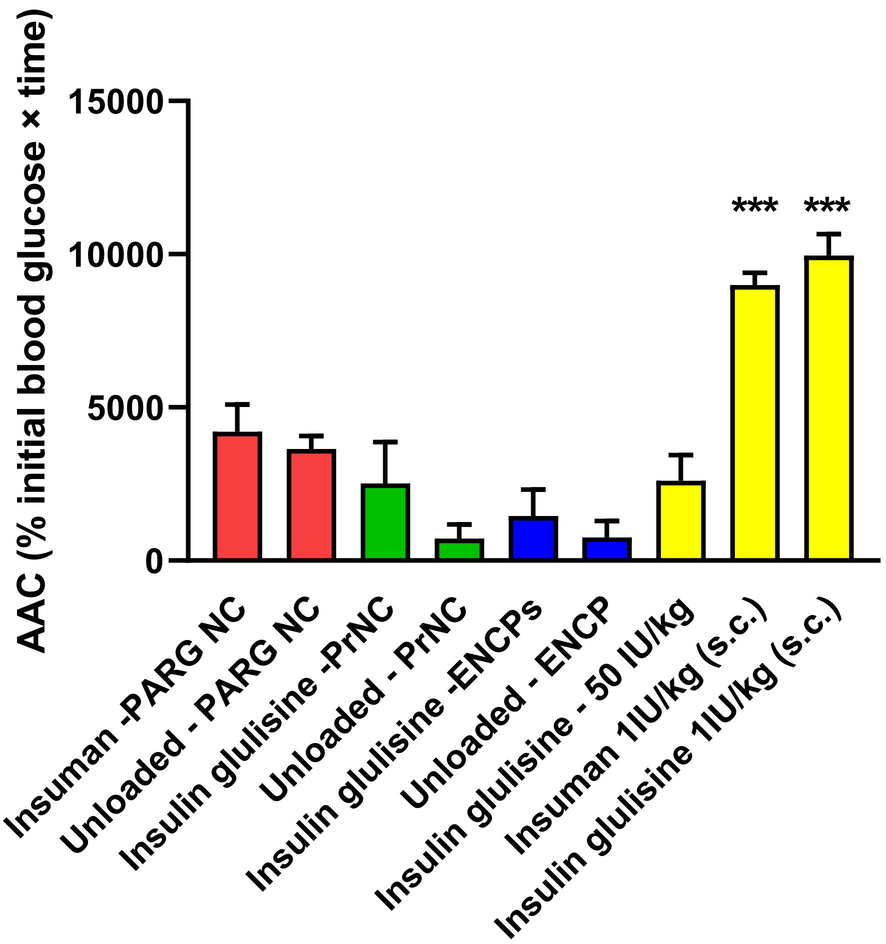

Fig. S9. Area above the blood glucose concentration – time curve (AAC) for the tested NP after jejunal instillation in rat.
Using the normalized blood glucose values illustrated in Fig. 7, the area above the concentration – time curve was calculated for the different tested NP. No NP showed a significantly different AAC when compared to its own unloaded control NP, devoid of insulin. In comparison, insulin after s.c. administration showed a large and significant increase in AAC.

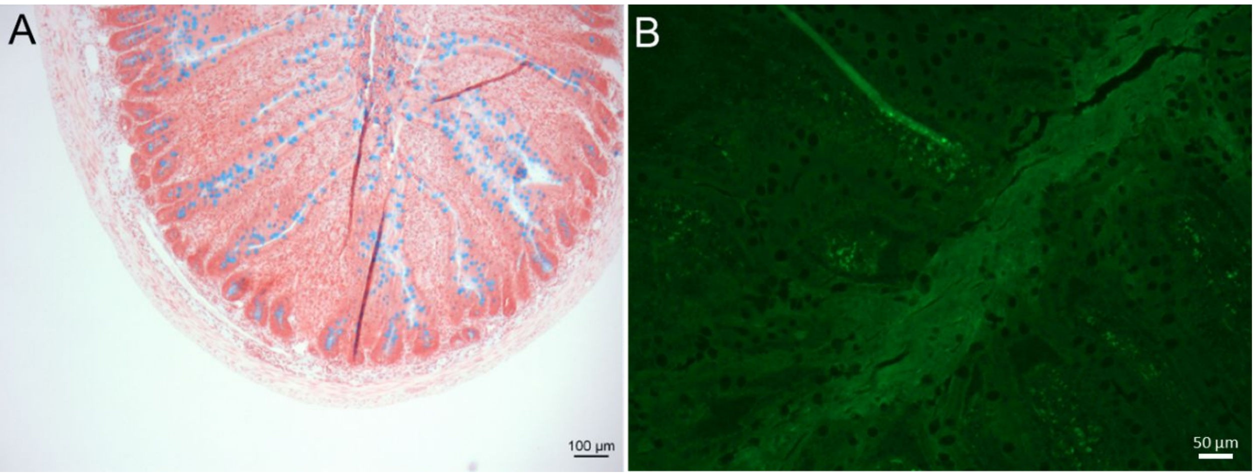

Fig. S10. Histology from rat *ex vivo* studies. A: Histological section of rat jejunum showing intact tissue after administration of NP to rat intestinal loops. Staining with haematoxylin/eosin and alcian blue. No distension of the intestinal lumen can be seen. **B:** Levels of autofluorescence in the FITC channel. Autofluorescence was consistently lower in TAMRA and DiD channels (not shown). Microscopy settings were the same as in Fig. 7F.
